# Contrasting movement strategies among juvenile albatrosses and petrels

**DOI:** 10.1038/srep26103

**Published:** 2016-05-18

**Authors:** Sophie de Grissac, Luca Börger, Audrey Guitteaud, Henri Weimerskirch

**Affiliations:** 1 Centre d’Etudes Biologiques de Chizé, UMR 7372 CNRS & Université de La Rochelle, Villiers en Bois, France; 2 UMR 9220 CNRS IRD ENTROPIE, Université de la Réunion, Saint Denis, France; 3 Department of Biosciences, College of Science, University of Swansea, Swansea, UK

## Abstract

Animal movement is a fundamental eco-evolutionary process yet the behaviour of juvenile animals is largely unknown for many species, especially for soaring seabirds which can range widely over the oceans at low cost. We present an unprecedented dataset of 98 juvenile albatrosses and petrels (nine species), tracked for the first three months after independence. There was a startling diversity within and among species in the type and scale of post-natal movement strategies, ranging from area-restricted to nomadic patterns. Spatial scales were clustered in three groups that ranged from <3000 km to >6000 km from the natal nest. In seven of the nine species, the orientation of flight paths and other movement statistics showed strong similarities between juveniles and adults, providing evidence for innate orientation abilities. Our results have implications for understanding the development of foraging behaviour in naïve individuals and the evolution of life history traits such as survival, lifespan and breeding strategy.

Juvenile and immature individuals play a key role in the dynamics of animal populations^1^, particularly in long-lived species for which they can represent up to 50% of the total population^2^. It is therefore essential to account for the specific traits and strategy of juveniles when modelling the fate of populations^3^. However, for most species, the behaviour and fate of juveniles remain almost unknown. In animals with parental care, the critical stage for juveniles is when they start to move and forage independently. At this stage, juveniles generally suffer high mortality, which is likely a result of low foraging efficiency or non-optimal morphology compared to that of adults^4^,^5^. How such naïve individuals navigate in their environment, especially for large scale dispersive movements such as migration or exploration, has been identified as a priority for research^6^.

The concept of dispersal, or natal dispersal, is often used to describe the movement of a juvenile leaving its natal grounds to breed elsewhere^7^. This therefore excludes the post-fledging movements of highly philopatric species, which return to breed at their natal colonies. However, as pointed out in recent studies^8^, dispersal includes the “movement of new fledglings in all directions with no inherent directional preferences”. These movements may, in time, lead to ‘true’ dispersal, in the most commonly used sense^7^, or to the birds’ return to their colony of origin.

Substantial individual variability has been documented in the dispersal behaviour of terrestrial animals^7^,^9^. Individuals move in heterogeneous environments, making their displacement landscape-dependant. Indeed, non-flying terrestrial animals are severely constrained by movement costs related to energy landscapes^10^,^11^. While birds are far less constrained, terrestrial birds still generally follow topographic features and landmasses during their migration or dispersal, and use specific sites for stop-overs^8^.The case of seabirds is very different, as adults have capacities for large scale movements over open, comparatively featureless oceans^12^,^13^. This is particularly true for procellariiform species (albatrosses and petrels), which can undertake extremely long migratory movements^14^,^15^. When juvenile petrels and albatrosses leave their nests for the first time, they have ‘the world as their oyster’, in that they can potentially fly at low metabolic costs, using winds to move in a vast, apparently homogenous, environment. These naive birds face no topographic constraints on where and when to move. Whilst the oceanic realm is certainly not a featureless environment for seabirds that are known to be sensitive to oceanographic features such as bathymetry, currents, wind zonation, eddies, or odours such as dimethyl sulphide^16^, the spatial scales are larger than in terrestrial habitats by many orders of magnitude^17^. This adds further interest to the question of how these animals forage in the marine environment.

In most seabird species, juveniles leave their natal grounds independently from their parents, and thus have to explore the environment and learn to forage entirely on their own. Existing studies on land birds have shown that juveniles may follow an innate navigation programme to some extent, while acquiring cues to improve their foraging proficiency^14^. The innate parts of juvenile movements can be similar to the movements of adults for some species^18^, while for others they may differ^19^,^20^ with respect to the timing^21^,^22^, track characteristics (e.g. sinuosity^23^) or route choice (e.g. less productive areas^4^,^22^,^24^). These differences in terrestrial birds have generally been explained by the lack of experience in juveniles^25^ leading to navigation errors. Though a lower competitiveness on foraging grounds in relation to adults^26^ may also have selected for endogenous post-fledging dispersal programmes that favour intra-specific spatial segregation. However, very limited data exist to allow a general understanding of the dispersal movements of juvenile seabirds within and across species. Without a strict endogenous programme and highly constraining landscape features, we may expect juvenile seabirds’ movements to vary considerably within and between species.

Here we present the first cross-species study of post-fledging movements in juvenile seabirds, comparing the large-scale movement strategies of juveniles from nine closely related procellariiform species during their first months at sea. The nine species, i.e. two species of great albatrosses, two species of mollymawk albatrosses, two species of sooty albatrosses, two species of giant petrels and one species of petrel (Table 1), constitute an ideal study system as they breed in the central Indian Ocean (Supplementary Fig. 1) and face similar environmental conditions when they leave their natal breeding sites. Adults of the nine species exhibit contrasting movement patterns during the inter-breeding period, i.e. when they are not central place foragers. They either show a typical migratory behaviour, i.e. moving regularly from breeding grounds to winter quarters and back^27^, ranging widely in the Southern Ocean with no specific destination, or remain near the breeding grounds throughout the year^20^,^28^,^29^,^30^. These species also show contrasting life history strategies, with great albatrosses and sooty albatrosses being longer lived than the other species, and juvenile survival being very different between species^31^,^32^,^33^. By comparing movement types and characteristics, we can test whether juveniles of each species follow a programmed route, whether their routes or destinations follow those of the adults or whether they differ in timing, directionality, sinuosity or targeted habitat type^8^,^23^,^34^. For example, rapid movement away from the natal colony or use of different areas than those used by adults may reduce intraspecific competition since it is generally assumed that competition between immatures and older birds leads to spatial segregation between age-classes^4^,^19^,^26^. Such competition may have consequences on individual fitness and population dynamics^4^,^35^.

The specific questions we want to address in this study are (1) Do closely related seabird species differ in their movements during the first months of independence at sea? (2) Do naïve juvenile birds have similar movement patterns to those of their parents? (3) Do the movement trajectories provide evidence of an innate navigation programme, implying unique fixed departure directions or direct orientation towards specific foraging areas instead of random movements? We discuss the implication that differences in movement behaviour may have on the evolution of life history components such as juvenile survival.

## Results

### Comparison between juveniles

#### General movement patterns

**Figure 1:**
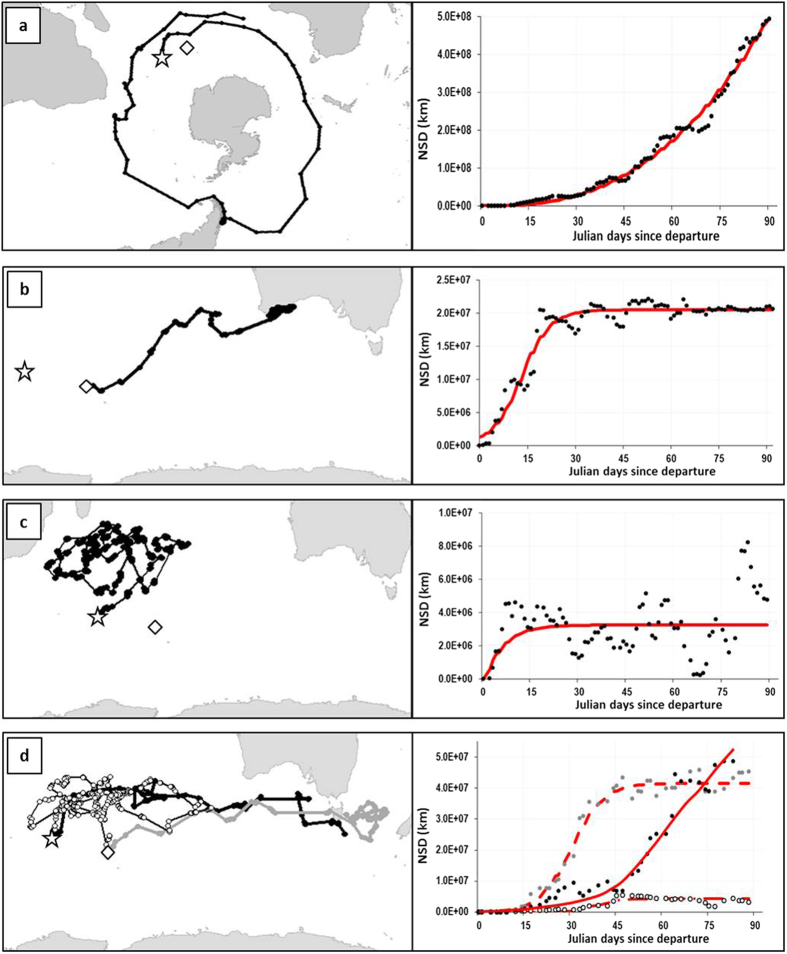
Typical juvenile trajectory patterns (left) and corresponding NSD curves (right, red lines are best NSD model fits) illustrated by (**a**) Northern giant petrel (nomadic type), (**b**) Black-browed albatross (half-migration type), (**c**) Sooty albatross (large-scale looping type best fitted here by a home-range NSD model). Panel (**d**) shows 3 different trajectory types of juvenile wandering albatross and NSD fits corresponding (red line) with nomadic type movement (black dots, full red line), half-migratory type movement (grey dots, dashed red line) and large-scale looping movement (white dots, dash-dot red line). White star and diamond are respectively Crozet and Kerguelen colonies. Maps have been generated with R^64^ and the “ggplot2” library^72^ and free-access coastline data (from *naturalearthdata.com*).

The Net Squared Displacement (NSD) method^36^ was used to model and identify typical large-scale movement types (Supplementary Methods 2-Fig. 1). We observed that juveniles showed striking interspecific differences in displacement behaviour in the first three months after leaving the natal colony. Movement types ranged from sedentary to nomadic patterns (Table 2, Fig. 1), and included migratory-like movements and a specific pattern that we named “large-scale looping type” (Fig. 1c). The majority of northern (67%) and southern (100%) giant petrels showed a typical nomadic-type movement with large dispersal distances (range at three months >6000 km, Table 2), and an eastward trajectory, with all individuals circumnavigating Antarctica within the first three months after leaving the natal colony (Table 2, Fig. 1a). Some individuals stopped for variable durations in specific areas before resuming their eastward movements and four individuals, for which the tracking time exceeded six months, completed a circumpolar movement and started a second one. In contrast, most juvenile mollymawks (100% of the yellow-nosed albatrosses and 86% of the black-browed albatrosses) and white-chinned petrels (100%) showed migratory-type movements fitted by the “half-migration” model. Specifically, the migratory–like movements of these species included a transit phase of 10–30 days with rapid directional movement, followed by a settling phase in a restricted area. Since these movements lacked the return trip, typical of true complete migration, during the 3–6 month study period, we named the corresponding model ‘half-migration model’ (e.g.: Fig. 1b). These movements were carried out over medium spatial scales (the range at three months was shorter than 6000 km but longer than 3000 km, Table 2, Fig. 1b). Juvenile sooty albatrosses and Amsterdam albatrosses showed more individual variability that included all displacement types on a small to medium spatial scale (range at three months <3000 km). Overall, the displacement types of these three latter species were not characterized by any clear prevalent displacement strategy, with most individuals remaining within a limited range from the colony through looping movements. We therefore grouped these individuals into a category named “large-scale looping type” (Fig. 1c).Wandering albatrosses showed an even higher intraspecific variability with some individuals moving over a small scale in the Indian Ocean and showing a looping behaviour (Fig. 1d: white dots), whereas others (60%, Table 2) moved further toward the Australian coast and/or into the Pacific Ocean (Fig. 1d: black and grey dots), adopting either half-migratory type movements or large-scale nomadic type movements. These differences in displacement lead juveniles of the nine species to relocate to three broad geographic areas, with small-scale dispersers (most sooty and great albatrosses) remaining in the central Indian Ocean, medium scale dispersers (mollymawk albatrosses, petrels and some wandering albatrosses) making some excursions out of the Indian Ocean, and large scale dispersers (giant petrels and some wandering albatrosses) leaving the Indian Ocean, with some birds undertaking entire circumpolar navigations.

#### Orientation at departure and further bearings

**Figure 2:**
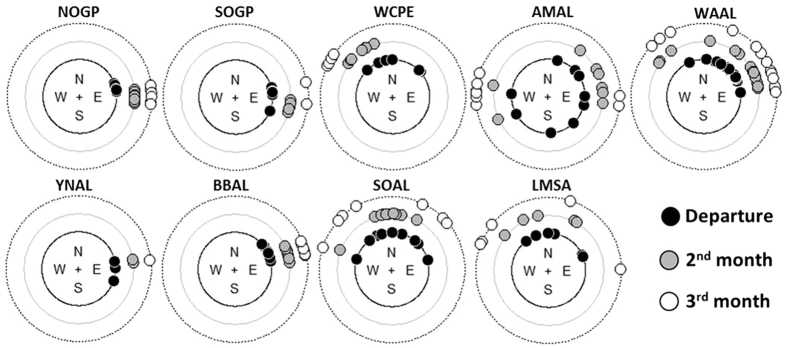
Orientations. Departure directions (black dots) and rhumb bearing of the position at 2 months (grey dots) and 3 months (white) with respect to the departure point, i.e. the birth colony.

Departure flight directions taken by juveniles from their natal colony (Fig. 2: black points) had two distinct patterns. **1:** An oriented trajectory with very little inter-individual variability in flight directions (within species, departure headings range was ≤π/4, indicating little variation in direction, Rayleigh test of uniformity: all P < 0.01,). This was the case for nomadic (giant petrels) and migratory type species (mollymawks and white-chinned petrel). These two groups of species also showed a high consistency in orientation with time, since the directionality of their position with respect to the colony at month 2 and 3 (Fig. 2: grey and white points) was similar to the direction taken at departure (bearings range <π/4) with a small shift toward east for white-chinned petrels. **2:** A high variability in departure heading toward east, west and north for the three species exhibiting large-scale looping movements, i.e. the two sooty albatrosses (heading toward north, π > range < π/2, Rayleigh test: P > 0.1) and Amsterdam albatrosses (range >π, Rayleigh test: P = 0.73). This group with non-oriented flights at departure showed no further consistency in orientation since positions were similarly scattered after two and three months. Finally, most wandering albatrosses had a restricted departure direction (direction range = 1.8 rad, just over π/2) toward NE, but the directionality of positions were not consistent beyond departure.

#### Sinuosity and daily distance travelled (DDT)

**Figure 3:**
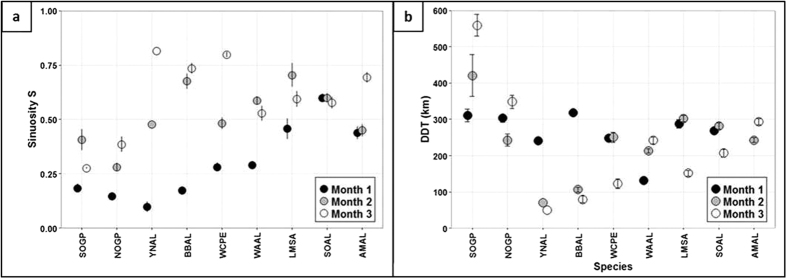
Sinuosity and daily distance travelled. (**a**) Average sinuosity of the trajectory during the first 15 days at sea of month 1 (black dots), month 2 (grey dots) and month 3 (white dots). (**b**) Average daily distance travelled (DDT) ± one standard error (bars) during the 15 first days of months 1 (black), 2 (grey) and 3 (white).

First, the paths of nomadic-type species (giant petrels) were typified by a lower sinuosity (S < 0.5) than other species (linear mixed model & post-hoc Tukey test: P < 0.05 when compared to all species except wandering albatross and yellow-nosed albatross, Fig. 3a & Supplementary Results 1). They also had the highest DDT (354 ± 58.9 km (SD)) of all species when averaged over the 3 months (linear mixed model & post-hoc Tukey test: P < 0.05 when compared to all species except light-mantled sooty albatross (P = 0.054 when compared to northern giant petrel), Fig. 3b and Supplementary Results 2). The DDT varied between individuals but showed no clear trend over time (linear mixed model & post-hoc Tukey test p-values in Supplementary Table 2). Second, migratory-type species performed trajectories that directly (low sinuosity <0.5) and rapidly (high DDT >240 km) took them from their natal colony to a restricted foraging area during the first month (i.e. during the transit phase). Once the destination was reached (during the 1^st^ (mollymawks) or 2^nd^ month (white-chinned petrel)) there was an increase in sinuosity (linear mixed model & post-hoc Tukey test: P < 0.05 except for yellow-nosed albatross, Supplementary Table 2) and a decrease in DDT (linear mixed model & post-hoc Tukey test: all P < 0.05, Supplementary Table 2). This phase corresponds to the settlement time, when the NSD curve reaches the asymptote. The reason why white-chinned petrels settled later than mollymawks (i.e. with transits lasting 30 to 40 days compared to 14 to 26 days in mollymawks) is that all individuals started heading north (Kerguelen colony) or north-east (Crozet colony) until they reached latitudes of 30°S (around the 9^th^ day), which corresponds to the latitude of Trade winds, i.e. easterly winds. Following this, individuals adopted a westerly heading to reach their final destination off the coasts of South Africa. Finally, the group with large-scale looping movements (sooty and Amsterdam albatrosses) had high orientation variability, medium sinuosity (close to 0.5 or higher) and medium DDT (241 ± 72 km (SD)) right from the first month, with no significant trend over the whole period (Fig. 3a, Supplementary Tables 2).

#### Distance to colony and habitats

**Figure 4:**
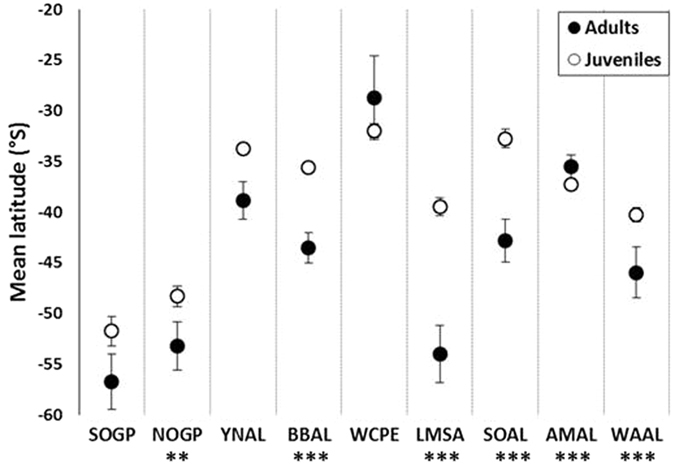
Latitudes. Mean latitude ± one standard error (bars) attained during the third month at sea by juveniles (white circles) and by adults during inter-breeding season (black circles). Significant differences between juveniles and adults are **P < 0.05 and ***P < 0.01 (Wilcoxon test).

During the first 15 days all species moved rapidly and directly away from the natal colony (mean range at 15 days = 1323 ± 786 km (SD)). After one month, nomadic giant petrels had reached a significantly longer range than the other species (linear mixed model and & post-hoc Tukey test: P < 0.05, Supplementary Table 3) and tended to stay in oceanic waters (Bathymetry <−3000 m, Table 3 & Supplementary Fig. 2). They encountered significantly cooler surface temperatures than other species (linear mixed model and & post-hoc Tukey test: P < 0.05, Supplementary Fig. 2) mainly because of their passage into the Pacific Ocean. Some northern giant petrels however, stopped for variable duration in neritic waters, for example in the productive Chilean Patagonian shelf (where chlorophyll *a* values >3 mg.m^3^). Migratory-type species rapidly increased their distance to the colony until they reached slope edges off Australia (mollymawks, mean bathymetry >−2000 m for both species, waters significantly shallower than that of other species (linear mixed model and & post-hoc Tukey test: P < 0.05, Table 3 & Supplementary Fig. 2) or oceanic waters along eastern South-African coasts in the Agulhas current area (white-chinned petrels, mean bathymetry = −3493 ± 391 m (SD), Table 3, Supplementary Fig. 2). White-chinned petrels foraged at lower latitudes than other species (Fig. 4) and encountered warmer surface temperatures than other species (Supplementary Fig. 2, significant difference only compared to giant petrels, wandering, black-browed and light-mantled sooty albatross; linear mixed model and post-hoc Tukey test: P < 0.05). The three looping species that remained within a 3000 km radius of their natal colony foraged almost exclusively in deep oceanic areas (respectively 100, 90% and 89% of individuals of the two sooty species and Amsterdam albatrosses, Table 3; mean bathymetry = −3793 ± 178 m (SD), see Supplementary Fig. 2). Amsterdam albatrosses encountered warmer waters at lower latitudes than others (Fig. 4, Supplementary Fig. 2). Finally, wandering albatrosses showed a strong individual variability with respect to the distance from the colony (from small to large dispersal range) and the habitat frequented, comprising both neritic (continental slopes or oceanic features like ridges) and oceanic areas (Table 3, Supplementary Fig. 2).

With respect to chlorophyll *a*, although there was a tendency for some species, such as northern giant petrels, black-browed, yellow-nosed and wandering albatrosses, to favour waters with higher chlorophyll *a* (Supplementary Fig. 2), the differences were only significant between northern giant petrels and three species: sooty albatrosses, Amsterdam albatrosses and white-chinned petrel (linear mixed model and & post-hoc Tukey test: P < 0.05)

### Comparison with non-breeding adults

When the breeding season ended, adult birds left the colony for an extended period, during the winter time for most species, returning to the colony 5–12 months later for a new breeding season. By fitting the migratory model to their trajectories, the NSD method identified three distinct at-sea dispersal scales for the adults of the nine species (Table 3). First, adult giant petrels stayed all year round close to their colony with low variation around an asymptotic range (Table 3). All the other species moved on average >3000 km from the colony (Table 3). Adult mollymawks moved to a distance of 5000–6000 km (medium scale) with little variation around the asymptotic range (Table 3). In other words, they moved rapidly to a specific zone, the wintering area, where they remained, with only small scale movements. This wintering area was the same for all individuals of the species and was located over neritic waters; nearby and over the Australian continental shelf. The two species of Sooty albatrosses and Amsterdam albatrosses had medium to long inter-breeding dispersal distances with higher range variations (larger wintering area scale) and a high variability within species (Table 3). These three species foraged mainly in oceanic habitats. White-chinned petrels were intermediate between the two latter groups and wandering albatrosses showed strong inter-individual variability with individuals belonging to the three groups and foraging both in neritic and oceanic waters (Table 3).

Finally, adults foraged at significantly higher latitudes than juveniles for six of the nine species (Wilcoxon test: P < 0.05 for northern giant petrel, black-browed, Amsterdam, wandering and both sooty albatrosses, Fig. 4). Adult and juvenile white-chinned petrels foraged at similar latitudes but different longitudes with adults foraging west of South-Africa (mean longitude = 14.72 ± 2.17°E (SD)) and juveniles foraging mostly east of South-Africa (mean longitude = 40.83 ± 1.11°E (SD)). There was no significant difference in latitude for southern giant petrels, but, as for white-chinned petrels, there was a longitudinal segregation between juveniles and adults caused by their different strategies (nomadic *vs* resident movement type).

## Discussion

Our study is the first to provide a comprehensive examination of juvenile movement strategies among seabirds. It shows clearly that (1) although species are closely related, the juveniles of the nine species differ extensively in their movement patterns when they leave their colony of origin, and (2) that for each species the movement types of juveniles in the first three months following independence are generally similar to those of wintering adults, except for the two species of giant petrel. In addition, movement patterns appear to be similar within most species, but show high variability for some species with individuals showing mixed strategies.

### Interspecific variability in movement strategies and comparison with adults

Overall, the juveniles of the nine species showed very large-scale post-fledging movements from basin-wide movements to circumpolar trips. A common behaviour found in all species is the preliminary rapid movement that takes all individuals away from the vicinity of their colony of origin. This behaviour may allow a reduction of competition with breeding adults present around the colony at this time, especially in the large albatrosses where breeding adults are present throughout the year. After this first transit phase, individuals used three very different main types of large-scale movement patterns.

Juveniles of the two species of mollymawks and white-chinned petrels all exhibited the same migratory strategy as the adults. Their trajectory parameters during the settlement phase are typical of foraging in a restricted area – suggesting an Area Restricted Search^37^. In mollymawks, destinations of juveniles partly overlapped with wintering zones of adults over the continental shelf or shelf-break. These species appear as ‘typical’ migratory species as the route to the wintering grounds corresponds to a narrow corridor used by all age groups, similar to that seen in certain land birds^8^,^18^,^38^. Analyses at finer scales may help to determine how juveniles and adults interact in specific overlapping winter feeding areas, e.g. in black-browed and yellow-nosed albatrosses, and how this affects juvenile survival and/or foraging skill acquisition.

Conversely, juvenile white-chinned petrels head north to exploit favourable Trade winds at low latitudes that allowed them to reach eastern Africa at low cost. In contrast, adults directly moved to the west to their over-winter site. This leads to segregation between adults and juveniles, at least during the first part of winter. Adults use mainly neritic productive waters of the Benguela current off western South-Africa, whereas juveniles use more oceanic features like oceanic ridges in sub-tropical relatively warm waters (Fig. 4 and Supplementary Fig. 2).

Circumpolar nomadism, i.e. flying with dominant winds toward East, allowed juvenile giant petrels to rapidly reach areas very distant from their colony and from resident wintering adults, resulting in an extreme case of segregation whereby juveniles do not overlap at all with adults^20^. Spatial segregation between age classes during all or part of the breeding cycle occurs in many species (e.g.^22^,^23^,^39^,^40^,^41^). It has been described for other procellariiform species like black-footed albatross^24^ or Manx shearwater^42^ and is generally explained by competitive exclusion of juveniles by experienced adults^4^ in the closest favourable foraging areas around the colonies. Whereas in many taxa segregation arises from size differences between young and adult^39^,^41^, it has been suggested that in monomorphic species with deferred sexual maturity, like seabirds, experience rather than size may drive segregation^42^. Competition between age classes may have important consequences for the dynamics of the population^26^ since it may affect the reproductive success and survival of individuals^8^,^43^. Naïve young individuals excluded actively from the best foraging places by adults can be forced to use suboptimal feeding areas^4^,^42^. Giant petrels are known for being particularly aggressive competitors^44^, mainly scavenging when around the colony. Thus we can assume that juveniles may not be competitive enough to access on land the seal and penguin carcasses or even fisheries waste used by adults. Juveniles may feed more on pelagic resources until they are able to compete with experienced birds. This creates ontogenic niche divergence and will probably result in a progressive shift in diet during the immaturity period. A similar pattern of spatial segregation was found between adult and juvenile giant petrels in the Southern Atlantic^45^, where juveniles moved out of the adults’ range after 30 days, to forage in productive upwelling areas that sustained many seabird species. In contrast to giant petrels from Crozet and Kerguelen, Southern Atlantic juvenile giant petrels, while spatially segregated, feed in a similar oceanic system as adults (shelf and shelf break)^45^.

The third strategy that involved large-scale looping movements at the scale of the Southern Indian Ocean was used by juvenile sooty albatrosses, Amsterdam albatrosses and some wandering albatrosses. Overlap between adult and juvenile of these species is probably reduced by the extensive size of the sector used in the large looping movements, and by differences in latitudes.

Although strategies differ extensively among albatrosses and petrels, they are consistent within genus. Each genus presents particular anatomic characteristics; different wing loading and wing shape that affect flight speed, manoeuvrability and energetic requirements at take-off or landing^46^. These differences are likely to affect dispersive abilities as well as foraging ecology^47^, an interesting aspect to investigate in further detail in future research. Albatrosses and petrels are known to make extensive use of wind to reduce energetic costs while flying^46^,^48^. In our study, fifty five percent of the individuals/species flew mainly with dominant winds, the Westerlies, at the beginning of their trip, and later on for giant petrels. Wind is probably a major factor affecting seabird dispersal and in some species it has been shown that young birds are also affected by other environmental conditions^18^,^25^ such as resource distribution^49^, e.g. signalled by dimethyl sulphide gradients (phytoplankton related odour)^16^.

### Individual variability

There were striking differences in individual variability within species, ranging from strongly consistent juvenile displacement types to species with a high degree of intra-specific variability. Most species belonged to the former category, whereas wandering albatrosses belonged to the latter, showing a mix of the three movement strategies with a very high individual variability in all parameters. Considering that all individuals encountered the same conditions at departure with essentially no movement constraints in an entirely unexplored environment, the low individual variability within long-range species that visit neritic waters (mollymawks, petrels) is particularly striking. Conversely, more oceanic species with shorter dispersal ranges tended to show more individual variability. This is particularly true for wandering albatross where individual variability is the highest. Those individual differences may be in part sex-specific^7^,^50^. However adults were similarly variable in migratory behaviour^7^,^51^, suggesting that variability in movement strategy is driven not only by external conditions but also by the traits and internal state of individuals (morphology, physiology, behaviour). In a relatively homogeneous landscape with few external movement constraints, internal factors may have a more pronounced effect on the large-scale movement patterns of juveniles and lead to higher individual variability than in coastal waters where a higher probability of interactions (due to topography, high bird density, fisheries) may affect movement costs and foraging decisions. Accordingly, despite high within-population variability, individual Atlantic puffins (*Fratercula arctica*) show high consistency among years in their own migratory routes^52^. The authors suggested that juvenile puffins may make long exploratory trips to different areas acquiring the experience needed to navigate their environment and find appropriate foraging zones. Since juvenile wandering albatrosses travelled across the whole Indian Ocean or further during their first year at sea, using mixed movement strategies, one might wonder if this could correspond to an exploratory behaviour. Individuals might acquire knowledge and progressively reduce the range of areas they visit and associated movement patterns until they adopt a foraging strategy that is consistent from year to year, as the adults do during sabbatical years throughout their life^51^.

### Innate navigation programme

While anatomy, physiology, competition and environmental conditions may strongly affect seabird post-fledging movement strategies^53^, our findings suggest that juveniles may also partly use an innate navigation programme when leaving their natal colony^54^. Unlike species for which juvenile migratory capacities are enhanced by the presence of adults^38^, juvenile procellariiformes are left by their parents before they fledge. However, we found that juvenile strategies were consistent from the start within species, and were similar to those of adults outside of the breeding season in most species. This suggests that juveniles first follow internal cues, especially for the timing and direction of departure, but also for their destination in the case of mollymawks and white-chinned petrels. The latter are striking examples since, in order to reach the targeted foraging ground west of their colony, all individuals first flew northwards to find favourable winds instead of following the Westerly flow or flying against it to reach directly the destination. This likely genetically encoded behaviour might have been selected to use the less expensive route in terms of energetics and cause the dispersion of juveniles to more distant feeding grounds^50^,^55^. In contrast to this innate behaviour, it has been suggested that young Atlantic puffins may rely more on a true learning process^42^. Overall, juvenile seabirds probably depend on both innate and acquired skills in different proportion depending on the species. For example, young Cory’s shearwaters innately follow the same general strategy as older birds, but progressively improve their migratory route with age^22^. Orientating in the apparently featureless environment of the pelagic ocean seems challenging, and the navigation cues, innate or acquired by experience and memory, are far from being clear^16^,^56^,^57^,^58^. This innate programme that drives juveniles on very specific routes may be a disadvantage in a context of rapid global change when birds may need to adapt to a shift in resources distribution, with unconditional fixed strategies probably being inferior to condition-dependent strategies^35^. In migrating birds several populations have retained their original route that has become sub-optimal in the context of climate change^59^. Interestingly, all these cases refer to species where the juveniles migrate independently from the adults and rely on their genetic programme for the first migration, such as our study species. There seems to be no such case of apparently sub-optimal routes among species where the juveniles accompany the adults on migration^59^. Thus, populations with independent juveniles may be less flexible in terms of migration or movement strategy, for example when facing long-term changes that need strategy adjustments.

### Link between dispersal strategies and life histories

The three main strategies used by juvenile albatrosses and petrels were found in three distinct groups: mollymawks and white chinned petrels, great and sooty albatrosses and giant petrels respectively. The first two groups have contrasting life history strategies independent of their size: mollymawks and white chinned petrels are shorter-lived species than the others, with annual breeding and high mortalities during the juvenile-immature stages, whereas great albatrosses and sooty albatrosses are longer lived biennial species with high survival during juvenile and immature phase^31^,^32^,^33^,^60^. Thus, the migratory strategy where juveniles and adults share the same winter zones over shelf areas may result in higher costs in terms of survival than the large looping strategy over oceanic waters. Moreover, oceanic species are generally longer lived than neritic species^61^. Giant petrels are particular in their dispersal strategies, they are shorter lived for their size but demographic data about the survival of juveniles is lacking^62^. Although future research with higher numbers of loggers is necessary to estimate the survival of juveniles during these first months, our results suggest a more general link between movement strategies, juvenile survival and life history strategies.

The findings of this study may have major implications for understanding the evolutionary mechanisms underlying the foraging behaviours of naïve individuals, and the link with the characteristics of energy and spatial landscapes. Similarly, our results emphasize the importance of linking movement strategies and population dynamics^1^,^35^. Thanks to the rapid development of new tagging technology, it is now possible to track multiple life history stages to study marine predator ecology but also to better understand why juveniles suffer higher costs during the early stage of their life.

## Methods

### Field work and telemetry

Field studies were carried out on the Crozet (46.2°S, 52.4° E), Kerguelen (49.4°S, 70.1°E) and Amsterdam (38.4°S, 77.3°E) islands, south western Indian Ocean (see map in Supplementary Fig. 1). All field procedures were approved by The Ethics Committee of IPEV and Comité de l′Environnement Polaire and were carried out in accordance with the approved guidelines. We tracked nine species of albatrosses and petrels belonging to different taxa: two species of great albatrosses (Amsterdam albatross *Diomedea amsterdamensis*, wandering albatross *D. exulans*), two species of Mollymawks albatrosses (Indian yellow-nosed albatross *Thalassarche carteri* and black-browed albatross *T. melanophris*), two species of sooty albatrosses (sooty albatross *Phoebetria fusca* and light-mantled sooty albatross *P. palpebrata*), two species of giant petrels (northern giant petrel *Macronectes halli* and southern giant petrel *M. giganteus*), and one species of petrel (white-chinned petrel *Procellaria aequinoctialis*) (Table 1). Instead of complete species names, species codes are used in tables and figures, as detailed in Table 1.

#### Juveniles

Between 2001 and 2014, 98 chicks (Table 1) were fitted with Argos satellite transmitters (Platform Terminal Transmitter, PTT 100, Microwave Telemetry, Columbia, USA) just before fledging. Units were fixed on the birds’ back feathers using adhesive tape and glue. The mass of devices (20–50 g) was always below the 3% of the body mass limit recommended for flying birds (Phillips *et al*. 2003). Transmitters were powered with solar panels and worked with duty cycle mode, 10 hON-24 hOFF, 18 hON- 54OFF or 12 hON-60 hOFF. Some of the juveniles were sexed using a molecular sexing method, others are of unknown sex. After data filtering and homogenization (Supplementary Methods 1) we obtained a total of 83 tracks, with 53 lasting at least 3 months and with an average of 0.65 ± 0.12 (SD) locations per day.

#### Adults

Adults from the same nine species and same sites were tracked during the non-breeding season, when they are no longer central place foragers. Birds were fitted with light-recording geolocators (GLS) weighting less than 3 g and attached to a leg band, beginning in 2000. GLS give positions twice per day with a low accuracy (median error of 180 km)^63^. To compare the overall large-scale movement patterns of juveniles and adults we randomly chose 10 complete adult tracks per species starting at the end of a breeding season and ending at the beginning of the next one. To compensate for the low accuracy of locations given by the GLS system we smoothed the trajectories by averaging locations on a 3-days’ time scale. Due to the low accuracy and low frequency of sampling we used adult data only for the comparative analysis of overall displacements and a global assessment of habitats used but not for the detailed trajectory analysis.

### Movement Analysis

We used two types of approach for analysing the movement data, both implemented using the R Software Environment^64^. **1:** The squared displacement method was used to identify and compare movement types within and between age classes and species^36^. **2:** Movement statistics commonly used for animal trajectory analysis were calculated to quantify detailed juvenile movement parameters and test the innate navigation hypothesis^65^.

### Comparative analysis of displacement

To identify and compare movement types within and between age classes and species, we used the squared displacement method^36^. The net squared displacement distance (NSD) is the squared beeline distance (calculated using Haversine formula) between the starting point (here, the natal colony of the birds) and each location of the trajectory. It is a fundamental statistic for movement studies^66^,^67^. We used the NSD method^36^ to identify and compare different broad movement types such as sedentary (“home range”), migratory and nomadic movement modes (Supplementary Methods 2-Fig. 1), and concurrently estimate general movement statistics such as distance covered (Supplementary Table 1). The approach is continuous time based and allows for the efficient analysis of unequally and irregularly sampled movement trajectories. Following^36^ we fitted the movement mode models (Supplementary Methods 2-Equation 1) using nonlinear mixed effects models (nlme in ‘nlme’ R library) and evaluated the support from the data for the different movement modes using the concordance criterion^68^. Before applying the goodness-of-fit measure we also checked that the estimated parameters (e.g. timing or distance of dispersal) were consistent with the spatio-temporal scales of the trajectory and discarded models that were inconsistent (Supplementary Table 1).

#### Juveniles

We applied NSD models to juvenile movement data covering the first 3 months after departure from the natal colony (see details in Supplementary Methods 2.1). Figure 1 shows typical juvenile trajectories along with the corresponding NSD curve and model fit. We identified a new movement type that we named “large-scale looping” (Fig. 1c, details in Supplementary Methods 2.2). To complete the information given by the NSD method, we calculated the distance from the colony reached at 3 months for an index of dispersion distance and classified species as follows: (1) small scale movements – birds which remained in the Indian Ocean, at a range shorter than 3000 km from the colony; (2) medium scale movements - birds which dispersed to the Australian or South-African coasts, i.e. between 3000 and 6000 km; and (3) large scale movements - birds which left the Indian Ocean and ranged farther than 6000 km.

#### Adults

For adult birds we applied the NSD method on the whole inter-breeding period, i.e. 5 to 12 months depending on the species. All adult trajectories were best fitted by the migratory model as adults always returned to the colony to breed. As for juveniles, we compared inter-breeding migration distances and displacements scale in the wintering areas along with the type of habitat used (neritic or oceanic) as detailed in Supplementary Methods 2.3.

### Trajectory parameters analyses

To quantify detailed juvenile movement parameters and test the innate navigation hypothesis, we calculated movement statistics commonly used for animal trajectory analysis^65^, i.e. orientation, daily distance travelled, sinuosity and range. For consistency, all parameters were averaged over 15 day time periods for the three first months of the trips for each individual. To aid visualization, however, we present only the first 15 days of each month in our figures, i.e. values for three periods of 15 days. Circular statistics for orientation analyses were computed with the R package “circular”^69^. For other parameters, we used linear mixed effect models (lme within ‘nlme’ R package^70^) with individual as random factor to take into account individual variability, followed by post-hoc Tukey tests (‘multcomp’ R package^71^) to test for temporal and interspecific differences (see lme & Tukey test results in Supplementary Results 1 & 2 and Supplementary Table 2). Differences between time periods or between species were considered significant at the P < 0.05 level in Tukey pair-wise comparison tests. For all models we visually checked that model assumptions were met. For Yellow-nosed albatrosses and other species after a certain amount of time, small sample sizes lead to unsatisfactory residual distribution and thus the tests for these species are to be considered with care (see supplementary Method 3 for details). Parameters calculated for each track were: the bearing taken at departure, the directionality of the bird with respect to the colony after two and three months, the sinuosity of tracks, the daily distance travelled and the mean latitude attained. Details for parameter calculations and statistics can be found in Supplementary Methods 3.

### Habitats used

To determine the conditions where juveniles settled in specific areas we examined the bathymetric characteristics of their last locations (neritic waters when depth >−3000 m, i.e. from lower continental slope to continental shelf, or oceanic area when over deeper waters), and compared this with adults’ broad habitat types (Supplementary Methods 2.3). Although it was not possible here to obtain accurate information on adult habitat, we extracted bathymetry, daily sea surface temperature (SST) and monthly chlorophyll *a* (CHL*a*) values for all juveniles’ trajectories. Environmental data were downloaded from the NOAA coast watch website (http://coastwatch.pfeg.noaa.gov/erddap/index.html). To allow comparison between species, bathymetry, SST and CHL*a* values have been summarised in Supplementary Fig. 2. As for trajectory parameters, differences between species have been tested with linear mixed models and Tukey post-hoc tests in order to take individual variability into account. We used all locations with environmental data available and took autocorrelation into account in the models. Conversely, the large inaccuracy of GLS locations did not permit to reach any conclusions about adult habitat characteristics.

## Additional Information

**How to cite this article**: Grissac, S. *et al*. Contrasting movement strategies among juvenile albatrosses and petrels. *Sci. Rep.* **6**, 26103; doi: 10.1038/srep26103 (2016).

## Supplementary Material

Supplementary Information

## Figures and Tables

**Table 1:** Dataset information.

Taxa	Species	Scientific name	Species code	Mass (kg)	Colony	Juveniles		Adults	
						N	Tracking time	N	Tracking time
**Great albatrosses**	Amsterdam albatross	*Diomedea amsterdamensis*	**AMAL**	8.0	A	11	123 ± 91 (21–280)	8	329 ± 48 (252–369)
	Wandering albatross	*Diomedea exulans*	**WAAL**	10.9	C, K	23	159 ± 89 26–379)	10	222.3 ± 13 (330–366)
**Mollymawks**	Yellow-nosed albatrosss	*Thalassarche carteri*	**YNAL**	2.6	A	4	48 ± 40 (3.0–94)	10	320 ± 31.6 (198–297)
	Black-browed albatross	*Thalassarche melanophris*	**BBAL**	3	K	12	69 ± 30 (15–96)	10	373 ± 26 (165–249)
**Sooty albatrosses**	Sooty albatross	*Phoebetria fusca*	**SOAL**	2.8	A, C	13	107 ± 79 (2–254)	10	310 ± 52 (174–375)
	Light mantled sooty albatross	*Phoebetria palpebrata*	**LMSA**	3.3	C, K	7	90 ± 60 (8–186)	10	136 ± 28 (273–366)
**Giant petrels**	Northern giant petrel	*Macronectes halli*	**NOGP**	5	C, K	10	118 ± 61 (44–227)	10	346 ± 15 (351–387)
	Southern giant petrel	*Macronectes giganteus*	**SOGP**	5	C	5	96 ± 65 (43–190)	10	322 ± 8 (120–144)
**Petrel**	White-chinned petrel	*Procellaria aequinoctialis*	**WCPE**	1.2	C, K	13	48 ± 42 (15–109)	10	271 ± 14 (303–336)
**Total**						**98**	**96 ± 37**	**88**	**292 ± 73**

Abbreviation used, mass, colony of origin and number of birds equipped with tracking duration (mean ± standard deviation (minimum–maximum)) by species, for juveniles and adults. Masses are only indicative for species and obtained from literature^73^. A, C and K are respectively Amsterdam, Crozet and Kerguelen Islands.

**Table 2:** Prevalence of different movement modes for juveniles of nine procellariiform species identified using the Net Squared Displacement (NSD) modelling method and movement scale at 3 months.

Taxa	Species	NSD models				Range at 3 months	Movement Scale
		Home-range	Nomad	Half migration	Migration		
**Giant petrels**	**NOGP**	0	66%	33%	0	17350 ± 2565	large
	**SOGP**	0	100%	0	0	23859 ± 2299	large
**Mollymawks**	**YNAL**	0	0	100%	0	4869	medium
	**BBAL**	0	14%	86%	0	5971 ± 1648	medium
**Petrels**	**WCPE**	0	0	100%	0	4117 ± 510	medium
**Sooty albatrosses**	**LMSA**	0	0	50%	50%	2221 ± 1421	small
	**SOAL**	57%	43%	0	0	1730 ± 702	small
**Great albatrosses**	**AMAL**	0	43%	57%	0	2943 ± 1525	small
	**WAAL**	0	33%	66%	0	4233 ± 2285	medium

**Table 3:** Dispersion distances and habitats visited by juveniles and adults.

Taxa	Species	Juveniles			Adults		
		Movement scale	Habitat	(%individuals)	Dispersion distance	Variation at asymptote	Habitat
**Giant petrels**	**NOGP**	large	O	(70%)	836 ± 143	357 ± 65	**N/O**
	**SOGP**	large	O	(80%)	1291 ± 186	364 ± 110	**N/O**
**Mollymawks**	**YNAL**	medium	N	(100%)	5004 ± 476	488 ± 265	**N**
	**BBAL**	medium	N	(81%)	3985 ± 328	291 ± 88	**N**
**Petrels**	**WCPE**	medium	N/O	(57%/43%)	5415 ± 328	605 ± 174	**N**
**Sooty albatrosses**	**LMSA**	small	O	(100%)	3862 ± 438	1084 ± 241	**O**
	**SOAL**	small	O	(90%)	4817 ± 1124	759 ± 430	**O**
**Great albatrosses**	**AMAL**	small	O	(89%)	3078 ± 966	712 ± 147	**O**
	**WAAL**	medium	O/N	(77%/23%)	5456 ± 457	1018 ± 847	**N/O**

All adults’ trajectories fitted by the migratory type NSD model, the dispersion distance is the distance to colony of the migratory model asymptote and the variation around the asymptote is calculated with the residuals of the model. Habitats are oceanic (O) and neritic (N).
