# Supplementary material for: Contrasting movement strategies among juvenile albatrosses and petrels
Source: Sci Rep. 2016 May 18;6:26103. doi: 10.1038/srep26103 (PMC4870643; doi:10.1038/srep26103)
Supplement: Supplementary Information [file srep26103-s1.pdf]

# Contrasting movement strategies among juvenile albatrosses and petrels

Sophie de Grissac* [a,b], Luca Börger [c], Audrey Guitteaud [a] and Henri Weimerskirch [a,b]

---

# Supplementary Information

## List of contents

**Supplementary Fig. 1.** Map of study colonies in the Indian Ocean……………………………………2

**Supplementary Methods 1.** Data preparation and tracking duration…………………………………4

**Supplementary Methods 2.** Net squared displacement analysis…………………………………………6

Methods 2. Fig.1. ……………………………………………………………………………………………………6

Methods 2. Equation 1. …………………………………………………………………………………7

Methods 2.1. NSD calculation for circumpolar trajectories ……………………………………7

Methods 2.2. Identification of the large-scale looping type movement …………………..8

Methods 2.3. Movement scale and habitat preference of adults……………………………8

**Supplementary Methods 3.** Trajectory parameter analyses……………………………………………10

**Supplementary Table 1.** Goodness of fit of NSD models fitted on individual trajectories…..12

**Supplementary Results 1.** Sinuosity: Comparison between species…………………………………16

**Supplementary Results 2.** Daily distance travelled: Comparison between species……………..18

**Supplementary Table 2.** Tukey test outputs for temporal variation within species in sinuosity and daily distance travelled…………………………………………20

**Supplementary Table.3.** Distance to colony: Comparison between species……………………..24

**Supplementary Fig.2.** Juveniles' habitat characterisation…………………………………………………25

Sophie de Grissac* [a,b], Luca Börger [c], Audrey Guitteaud [a] and Henri Weimerskirch [a,b]

## Supplementary Fig.1. Map of study colonies in the Indian Ocean

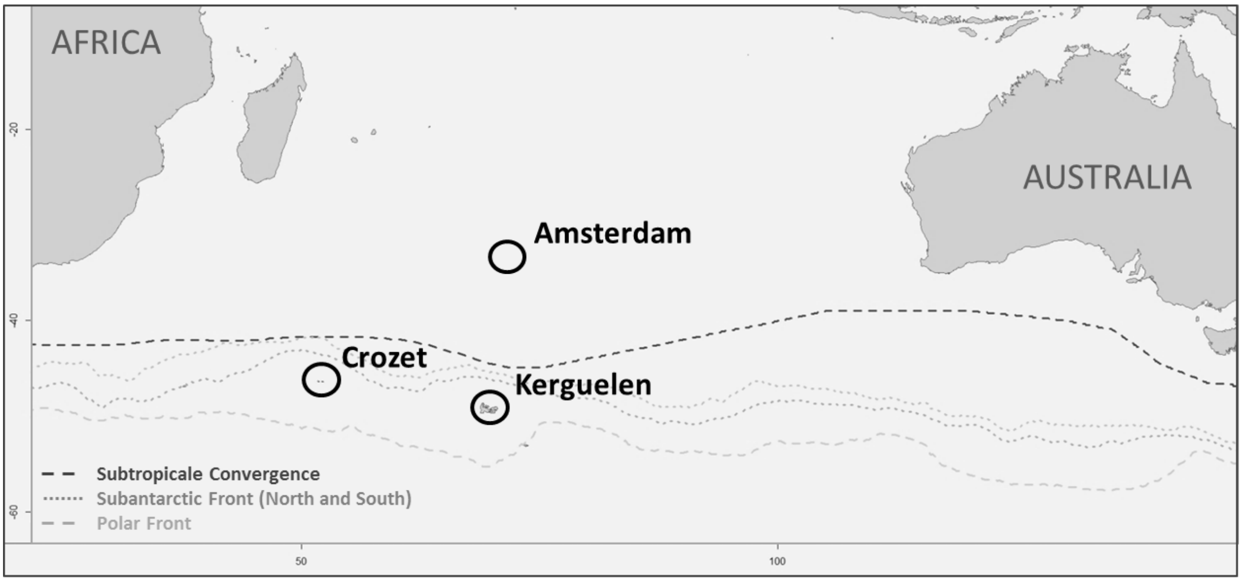


The map has been generated with R v3.2.3 (R Core Team, 2014) and the 'ggplot2' (Wickam, 2009) library and free-access coastline data (from naturalearthdata.com). Polar and sub Antarctic Front have been calculated from Aviso products (Altimetric Sea Level Anomaly of Sallee *et al.*,2008) available online at http://ctoh.legos.obs-mip.fr/applications/mesoscale/southern-ocean-fronts. Subtropical Convergence data come from Belkin & Gordon (1996).

## References

Belkin, I. M., & Gordon, A. L. Southern Ocean fronts from the Greenwich meridian to Tasmania, *J. Geophys. Res.* **101**(C2), 3675-3696 (1996).

R Core Team (2015). R: A language and environment for statistical computing. R Foundation for Statistical Computing, Vienna, Austria. URL http://www.R-project.org/.

Sallee, J.B., Speer, K. & Morrow, R. Southern Ocean fronts and their variability to climate modes, *Journ. of Climate*, **21**, pp. 3020-3039 (2008).

Wickham, H. in *ggplot2: Elegant graphics for data analysis*, (Springer, New York, 2009).

# Supplementary Methods 1. Telemetry data preparation and tracking duration

## Telemetry data preparation

For juveniles, all Argos locations (classes A, B, 0, 1 to 3) were used. Unrealistic positions were filtered out by removing those with an estimated speed above 90km/h (McConnell *et al.* 1992). Because of the use of duty cycled transmitters with varying duty-cycles, significant disparities in location frequency occurred between individuals. In order to make comparisons between individuals with similar location frequencies, we resampled the time series to obtain a maximum of one location every 10h, removing intermediate locations when necessary. This resulted in tracks with a mean of 0.65±0.12 locations per day with time between locations varying from 10 to 72h. Some trajectories were discarded because the tracking time was too short or locations were too infrequent because of tag malfunction, giving a total of 83 tracks including 53 tracks lasting at least 3 months used for the analyses.

## Tracking duration

We chose to analyse only the 3 first months of juvenile's trajectories because more than 50% of juvenile's tags stopped before 100 days. Tags were very similar for most species, and there was no reason for them to differ in duration of transmission or likelihood of failure. Yet when looking closely at the duration of transmission of tags, it was obvious it differed between species, and that the smaller the species, the shorter the transmission. This may be either because the mortality of smaller species is higher, or because the attachment of tags on smaller species is less efficient since the tags used are basically of the same size. Since back

feathers on smaller species are smaller and more likely to be detached after a certain time, this latter hypothesis is the most likely.

## References

McConnell, B. J., Chambers, C., & Fedak, M. A. Foraging ecology of southern elephant seals in relation to the bathymetry and productivity of the Southern Ocean. *Antarctic Science*, **4,** 393–398 (1992a).

# Supplementary Methods 2. Complement to methods: net squared displacement analyses

**Methods 2. Fig.1.** Typical NSD (Net Square Displacement from departure colony) over time curve shapes of the four movements types, adapted from (Bunnefeld *et al.* 2011).

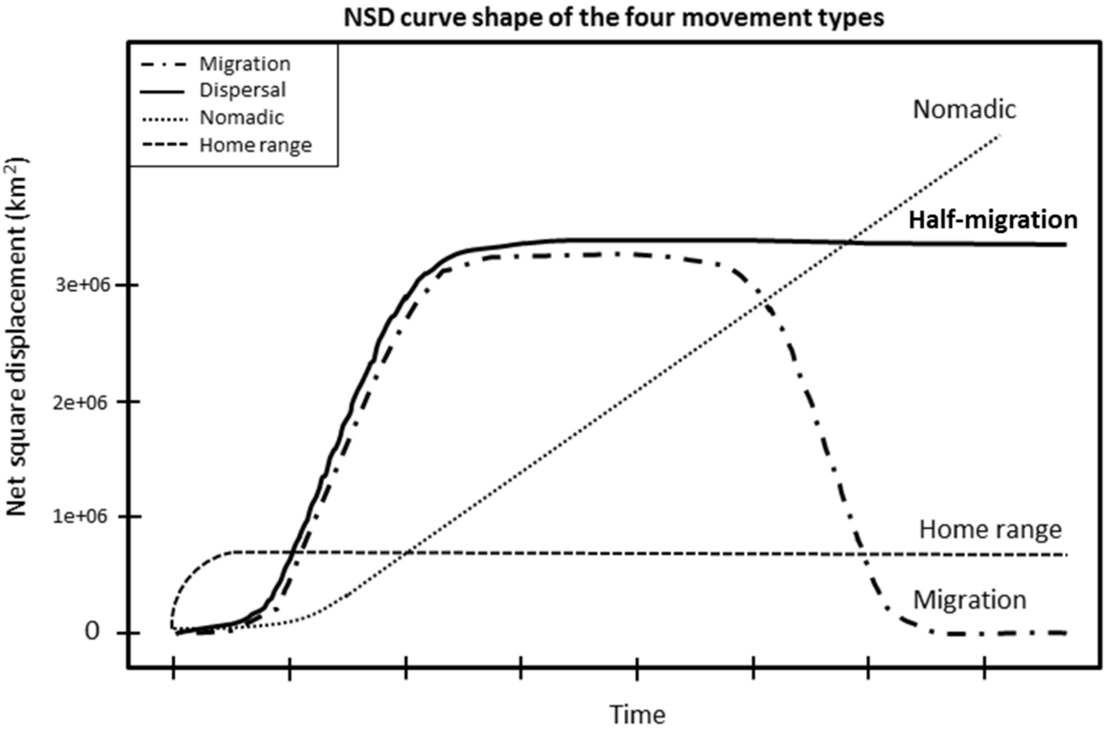


The model we termed here "half-migration" corresponds to the "Half-migration" model of Bunnefeld *et al.* (2011) and Börger & Fryxell (2012). We renamed it for consistency with juvenile and adult strategies and because juveniles will eventually return to their natal grounds completing, with time, a "true migratory" trip.

**Methods 2 - Equation 1.** Equations of the four NSD models (Borger & Fryxell 2012). *t* is the time since start. D and *b* are the diffusion constant and exponent. *Asym* is the asymptote at the settlement ($km^2$) and quantifies the movement distance. θ is a parameter for the inflexion point, in units of time since start (*t*), and thus models the timing of the transience phase. ϕ is a scale parameter on the time axis governing the shape of the curve.

$$NSD^2 = \mathrm{D} \times t^b$$

Home-range

$$NSD^2 = \mathrm{Asym} \times (1 - e^{\alpha t})$$

Nomadism

$$NSD^2 = \frac{Asym}{1 + exp\left(\frac{\theta - t}{\varphi}\right)}$$

Half-migration

$$NSD^2 = \frac{Asym}{1 + exp\left(\frac{\theta_a - t}{\varphi_a}\right)} + \frac{-Asym}{1 + exp\left(\frac{\theta_b - t}{\varphi_b}\right)}$$

Migration

.

**Methods 2.1. NSD calculation for circumpolar trajectories.**

For species making multiple circumpolar trips by flying continuously in the same direction (Figure 2.a left), the NSD values increase until they reach the longitude opposite to their departure colony (colony longitude – 180°) then they decrease until they reach the departure longitude. To avoid this artefact in distance calculations, we modified the calculation of the NSD in order to keep the continuously increasing flight distance characteristic of the trajectory. Specifically, the NSD was calculated as if the birds kept flying away from their departure point; see Fig.2.a, right panel, in the main text. For comparison, Fig 2.b, left panel, main text, shows a trajectory typical of the half-migration type model.

### Methods 2.2. Identification of the large-scale looping type movement.

We identified a movement mode not originally considered in the (Borger & Fryxell 2012) approach, termed large-scale looping movement. It is characterized by individuals remaining in the Indian Ocean for all three months after leaving the natal colony, but with large and frequent foraging loops within the Indian Ocean, reaching up to >1000km distance from the natal colony, then coming back again close to the departure area (e.g. Fig. 2c).

### Methods 2.3. Movement scale and habitat preference of adults.

In order to characterize the large scale strategy of adults we used the asymptote distance from colony (i.e. migration distance) given by the migratory NSD model fitted to each trajectory as an estimation of the inter-breeding movement scale. The residuals of the model (variation of the NSD around the asymptote) give an estimation of displacement and wintering area scales. We determined the bathymetric characteristics of the wintering areas (neritic/oceanic) by looking broadly at the trajectories but GLS data do not permit to calculate precise bathymetric characteristics of the zones foraged.

### References

Börger, L. *et al*. Quantifying individual differences in dispersal using net squared displacement. *Dispersal Ecol. Evol.* 222–230 (2012).

Bunnefeld, N., Börger, L., van Moorter, B., Rolandsen, C.M., Dettki, H., Solberg, E.J. & Ericsson, G. A model-driven approach to quantify migration patterns: individual, regional and yearly differences. *Journal of Animal Ecology*, **80**, 466–476 (2011).

# Supplementary Methods 3. Complement to methods: trajectory parameters analyses

## Orientation

Absolute flight directions were estimated as the loxodromic (or rhumb) flight bearings in relation to the geographic North. We first calculated heading at departure, arbitrarily chosen as the mean of the absolute directions taken during the first ten days since departure. Then we calculated the bearing of the positions at two and three months, i.e. the loxodromic direction of the position on the first day of each month from the colony location. For circumpolar trajectories, we corrected for the artefact of direction reversal once the longitude opposite to the departure point is crossed (i.e. > 180°) by setting the bearings as “bearing – pi”.

## Sinuosity

The large scale sinuosity of the trajectories was calculated over 15 day time windows as follows: $\mathbf{S = 1 - D_a/D_b}$ , with $D_a$ the beeline distance between loc($t_1$) and loc($t_n$) and $D_b$ the real distance travelled between loc($t_1$) (day 1 of the time window) and loc($t_{15}$) (day 15 of the time window).

## Daily distance travelled

At this scale, the DDT (distance travelled per 24 hours) we calculated integrates flight speed, time-spent on water and small scale sinuosity. Distance to colony: We calculated the loxodromic distance to the natal colony from each location of the trajectory (range) and computed the mean and the standard deviation for 3 time periods for each individual (1, 2 and

3 months after departure). The standard deviation gives an indication of the space-scale dispersion of locations. An individual with a low range standard deviation tends to stay in a small area around the mean range value whereas a bird with a high range standard deviation tends to forage in a large area around the mean range value or tends to keep going away from the starting point.

**Mean latitude**

We tested for differences in mean latitude of the trajectory of juveniles and adults using the Wilcoxon test. As there were no significant differences within species between birds from different colonies (Wilcoxon test: $P > 0.1$), for adults and juveniles, we did not separate individuals by colony of origin.

**Note about test significance and sample size**

With only one juvenile yellow-nosed albatross tracked more than three months, tests for this species cannot be reliable and in those cases, prior assumptions to modelling were often not respected. The same is true for southern giant petrels after 1.5 month (only two individuals left), Light-mantled sooty albatrosses after 2 month and white-chinned petrels for the last period (after 2.5 month).

## Supplementary Table 1. Goodness of fit values of NSD models fitted on individual 3 months trajectories.

Goodness of fit values are calculated for the 4 NSD model types applied to each juvenile. The null model ($NSD^2$ = constant) is used as a reference. Models for which the parameter outputs (asymptote timing and distance) are out of the data range are pointed out by an asterisk. The best model is the one with the highest GOF value and consistent parameter outputs. The asymptote distance returned by the best model is given in km along with the distance from colony at the end of the 3 months corresponding to a dispersion distance category (see Methods).

### Northern giant petrel (NOGP)

| Trip ID | Null | Home-Range | Half-migration | Nomad | Migratory | Best model | Colony | Asymptote distance | Range at 3 months | Dispersion scale |
|---|---|---|---|---|---|---|---|---|---|---|
| 87436 | 0.000 | 0.551 | **0.992** | 0.923 | 0.725* | Half-migration | Kerguelen | 14948 | 14305 | large |
| 87470 | 0.000 | 0.571* | 0.961 | **0.970** | 0.610* | Nomad | Crozet | - | 20555 | large |
| 87471 | 0.000 | 0.555 | **0.995** | 0.908 | 0.733* | Half-migration | Crozet | 16845 | 15841 | large |
| 87476 | 0.000 | 0.613* | 0.959* | **0.954** | 0.429* | Nomad | Crozet | - | 17146 | large |
| 87478 | 0.000 | 0.644* | 0.986* | **0.983** | 0.526* | Nomad | Kerguelen | - | 15895 | large |
| 87480 | 0.000 | 0.560* | 0.896* | **0.916** | 0.589* | Nomad | Kerguelen | - | 20365 | large |

### Southern giant Petrel (SOGP)

| Trip ID | Null | Home-Range | Half-migration | Nomad | Migratory | Best model | Colony | Asymptote distance | Range at 3 months | Dispersion scale |
|---|---|---|---|---|---|---|---|---|---|---|
| 87466 | 0.000 | 0.219* | 0.988* | **0.990** | 0.106* | Nomad | Crozet | - | 25411 | Large |
| 87474 | 0.000 | 0.297* | 0.987* | **0.994** | 0.066* | Nomad | Crozet | - | 22251 | large |

**Yellow-nosed albatross (YNAL)**

| Trip ID | Null | Home-Range | Half-migration | Nomad | Migratory | Best model | Colony | Asymptote distance | Range at 3 months | Dispersion scale |
|---|---|---|---|---|---|---|---|---|---|---|
| 103667 | 0.000 | 0.886 | **0.939** | 0.773 | 0.927* | Half-migration | Amsterdam | 4393 | 4869 | Medium |
| 79356 | 0.000 | 0.831 | **0.991** | 0.738 | 0.837* | Half-migration | Amsterdam | 3436 | 3486 | medium |

**Black-browed albatross (BBAL)**

| Trip ID | Null | Home-Range | Half-migration | Nomad | Migratory | Best model | Colony | Asymptote distance | Range at 3 months | Dispersion scale |
|---|---|---|---|---|---|---|---|---|---|---|
| 103665 | 0.000 | 0.751 | **0.970** | 0.850 | 0.855* | Half-migration | Kerguelen | 4530 | 4555 | medium |
| 103666 | 0.000 | 0.971* | 0.976* | **0.980** | 0.690* | Nomad | Kerguelen | - | 9170 | large |
| 132031 | 0.000 | 0.562 | **0.975** | 0.780 | 0.715* | Half-migration | Kerguelen | 5120 | 5144 | medium |
| 132032 | 0.000 | 0.793 | **0.987** | 0.886 | 0.874* | Half-migration | Kerguelen | 5674 | 5836 | medium |
| 132034 | 0.000 | 0.706 | **0.990** | 0.846 | 0.830* | Half-migration | Kerguelen | 5470 | 5428 | medium |
| 132038 | 0.000 | 0.811 | **0.988** | 0.779 | 0.679* | Half-migration | Kerguelen | 5717 | 5713 | medium |
| 132039 | 0.000 | 0.917 | **0.983** | 0.903 | 0.782* | Half-migration | Kerguelen | 5386 | 5456 | medium |

**White-chinned petrel (WCPE)**

| Trip ID | Null | Home-Range | Half-migration | Nomad | Migratory | Best model | Colony | Asymptote distance | Range at 3 months | Dispersion scale |
|---|---|---|---|---|---|---|---|---|---|---|
| 67413 | 0.000 | 0.852* | **0.963** | 0.885 | 0.403* | Half-migration | Crozet | 4611 | 4806 | medium |
| 87481 | 0.000 | 0.880* | **0.904** | 0.885 | 0.713* | Half-migration | Kerguelen | 4638 | 3902 | medium |
| 87482 | 0.000 | 0.951* | **0.960** | 0.950 | 0.741* | Half-migration | Kerguelen | 4154 | 3951 | medium |
| 87485 | 0.000 | 0.924* | **0.953** | 0.933 | 0.764* | Half-migration | Kerguelen | 3811 | 3650 | medium |

**Light-mantled albatross (LMSA)**

| Trip ID | Null | Home-Range | Half-migration | Nomad | Migratory | Best model | Colony | Asymptote distance | Range at 3 months | Dispersion scale |
|---|---|---|---|---|---|---|---|---|---|---|
| 87459 | 0.000 | 0.6761* | 0.809 | 0.669 | **0.87** | Migratory | Crozet | 1786 | 1550 | Small |
| 87460 | 0.000 | 0.064 | 0.109 | 0.0068* | **0.886** | Migratory | Crozet | 1658 | 1632 | Small |
| 87487 | 0.000 | 0.100 | **0.14** | 0.0085* | 0.913* | Half-migration | Kerguelen | 2594 | 3253 | Medium |
| 87489 | 0.000 | 0.086 | **0.201** | 0.003* | 0.862* | Half-migration | Kerguelen | 2636 | 1225 | small |

**Sooty albatross (SOAL)**

| Trip ID | Null | Home-Range | Half-migration | Nomad | Migratory | Best model | Colony | Asymptote distance | Range at 3 months | Dispersion scale |
|---|---|---|---|---|---|---|---|---|---|---|
| 34137 | 0.000 | **0.149** | 0.110 | 0.094 | 0.127* | Home-range | Amsterdam | 1123 | 1356 | small |
| 34145 | 0.000 | **0.024** | 0.022 | -0.006 | 0.123* | Home-range | Amsterdam | 1270 | 1369 | small |
| 66209 | 0.000 | 0.130 | 0.094 | **0.233** | -0.076* | Nomad | Crozet | - | 2183 | small |
| 67410 | 0.000 | 0.381 | 0.406 | **0.523** | 0.493* | Nomad | Crozet | - | 1908 | small |
| 67411 | 0.000 | **0.086** | 0.019 | -0.169 | 0.299* | Home-range | Crozet | 1664 | 878 | small |
| 87465 | 0.000 | 0.262 | 0.253 | **0.349** | 0.353* | Nomad | Crozet | - | 2853 | small |
| 93725 | 0.000 | **0.191** | 0.114 | 0.033 | 0.189* | Home-range | Amsterdam | 1552 | 1237 | small |

**Amsterdam albatross (AMAL)**

| Trip ID | Null | Home-Range | Half-migration | Nomad | Migratory | Best model | Colony | Asymptote distance | Range at 3 months | Dispersion scale |
|---|---|---|---|---|---|---|---|---|---|---|
| 112947 | 0.000 | 0.236* | **0.515** | 0.241 | 0.354* | Half-migration | Amsterdam | 1840 | 2158 | Small |
| 112948 | 0.000 | 0.236* | **0.779** | 0.502 | 0.637* | Half-migration | Amsterdam | 3114 | 447 | Small |
| 112950 | 0.000 | 0.281* | 0.752* | **0.719** | 0.735* | Nomad | Amsterdam | 3579 | 2825 | Medium |
| 40660 | 0.000 | 0.297* | 0.971* | **0.590** | 0.480* | Nomad | Amsterdam | 3935 | 3141 | Medium |
| 53936 | 0.000 | 0.435* | 0.703 | 0.710 | 0.686* | Half-migration | Amsterdam | 2841 | 2656 | Small |
| 53937 | 0.000 | 0.170* | **0.963** | 0.887 | 0.938* | Half-migration | Amsterdam | 4300 | 4292 | Medium |
| 53938 | 0.000 | 0.163* | 0.973* | **0.965** | 0.967* | Nomad | Amsterdam | 5221 | 5278 | medium |

**Wandering albatross (WAAL)**

| Trip ID | Null | Home-Range | Half-migration | Nomad | Migratory | Best model | Colony | Asymptote distance | Range at 3 months | Dispersion scale |
|---|---|---|---|---|---|---|---|---|---|---|
| **11817** | 0.442 | 0 | 0.316 | **0.453** | 0.456* | Nomad | Crozet | - | 2231 | small |
| **1391** | 0.836 | 0 | 0.799 | **0.949** | 0.783* | Nomad | Crozet | - | 4905 | large |
| **25751** | 0.280 | 0 | 0.154 | **0.320** | 0.167* | Nomad | Crozet | - | 584 | small |
| **38557** | 0.630 | 0 | **0.913** | 0.590 | 0.706* | Half-migration | Crozet | 5993 | 5693 | large |
| **38558** | 0.794 | 0 | **0.924** | 0.825 | 0.885* | Half-migration | Crozet | 5970 | 6278 | large |
| **38559** | 0.088 | 0 | **0.373** | 0.153 | 0.071* | Half-migration | Crozet | 1466 | 748 | small |
| **78345** | 0.240 | 0 | **0.527** | 0.184 | 0.479* | Half-migration | Kerguelen | 3957 | 2052 | medium |
| **78346** | 0.906 | 0 | **0.993** | 0.903 | 0.979* | Half-migration | Kerguelen | 5961 | 6105 | large |
| **78348** | 0.919 | 0 | **0.991** | 0.908 | 0.990* | Half-migration | Kerguelen | 6497 | 6719 | large |

| | | | | | | | | | | |
|---|---|---|---|---|---|---|---|---|---|---|
| **78349** | 0.584 | 0 | **0.712** | 0.549 | 0.674* | Half-migration | Kerguelen | 3925 | 3797 | medium |
| **78350** | 0.726 | 0 | **0.899** | 0.686 | 0.882* | Half-migration | Kerguelen | 4772 | 3871 | medium |
| **78351** | 0.906 | 0 | **0.977** | 0.944 | 0.951* | Half-migration | Kerguelen | 6633 | 6846 | large |
| **78352** | 0.319* | 0 | 0.964* | 0.959* | 0.241* | No fit | Kerguelen | - | 5974 | large |
| **8959_01** | 0.309 | 0 | 0.283 | **0.324** | 0.293* | Nomad | Crozet | - | 1436 | small |
| **8959_02** | 0.782* | 0 | 0.974 | **0.982** | 0.719* | Nomad | Crozet | - | 6956 | large |
| **8960_01** | 0.619 | 0 | **0.913** | 0.896 | 0.532* | Half-migration | Crozet | 2148 | 2667 | medium |
| **8960_02** | 0.915 | 0 | **0.976** | 0.940 | 0.953* | Half-migration | Crozet | 6847 | 6977 | large |
| **9059** | 0.510 | 0 | **0.855** | 0.761 | 0.443* | Half-migration | Crozet | 1912 | 2530 | medium |

## Supplementary Results 1. Sinuosity: comparison between species

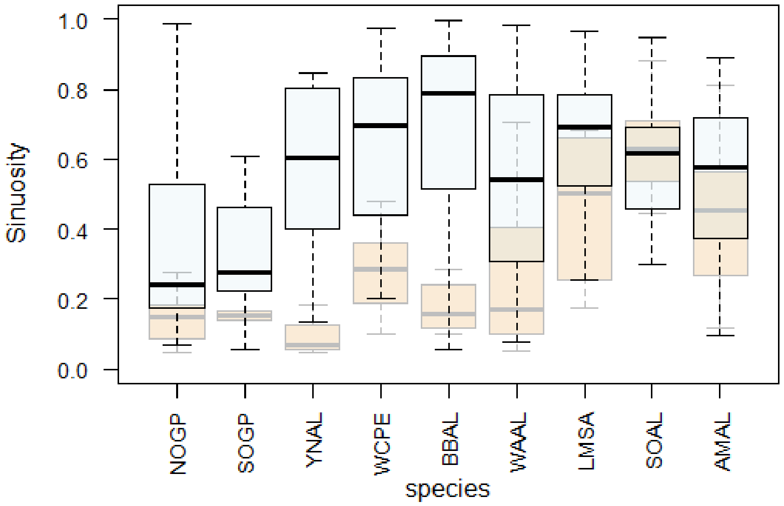


Boxplots of the sinuosity by species, during the first 15 days (cream & grey boxes) and averaged over the rest of the trajectories (15 days to 3rd month, blue & black boxes). Horizontal bars are the median, box limits are upper and lower quartiles, whiskers represent dispersion.

**Linear mixed model results:**

Model: Sinuosity ~ species + random = ~1 | individual

| SPECIES | **Value** | **Std.Error** | **DF** | **t-value** | **p-value** |
|---|---|---|---|---|---|
| **(Intercept: NOGP)** | 0.326 | 0.039 | 287 | 8.293 | 0 |
| **SOGP** | -0.027 | 0.071 | 73 | -0.387 | 0.6996 |
| **YNAL** | 0.121 | 0.087 | 73 | 1.391 | 0.1685 |
| **WCPE** | 0.235 | 0.060 | 73 | 3.886 | 0.0002 |
| **BBAL** | 0.257 | 0.055 | 73 | 4.719 | 0 |
| **WAAL** | 0.150 | 0.050 | 73 | 3.020 | 0.0035 |
| **LMSA** | 0.279 | 0.069 | 73 | 4.040 | 0.0001 |
| **SOAL** | 0.272 | 0.055 | 73 | 4.971 | 0 |
| **AMAL** | 0.201 | 0.056 | 73 | 3.619 | 0.0005 |

Notes: Treatment contrasts were used, hence the parameter estimate for the Intercept corresponds to the first species, here NOGP, ('reference value'), whilst all other values are the estimated differences compared to the first ('reference') species.

**Post-hoc Tukey test p-values:**

| SPECIES | **NOGP** | **SOGP** | **YNAL** | **WCPE** | **BBAL** | **WAAL** | **LMSA** | **SOAL** | **AMAL** |
|---|---|---|---|---|---|---|---|---|---|
| **NOGP** | | | | | | | | | |
| **SOGP** | 1 | | | | | | | | |
| **YNAL** | 0.8951 | 0.835 | | | | | | | |
| **WCPE** | <0.01 | 0.0122 | 0.9378 | | | | | | |
| **BBAL** | <0.01 | <0.01 | 0.8075 | 1 | | | | | |
| **WAAL** | 0.0599 | 0.1455 | 1 | 0.8187 | 0.3701 | | | | |
| **LMSA** | <0.01 | <0.01 | 0.7701 | 0.9996 | 1 | 0.5171 | | | |
| **SOAL** | <0.01 | <0.01 | 0.7046 | 0.9994 | 1 | 0.2083 | 1 | | |
| **AMAL** | <0.01 | 0.0306 | 0.9909 | 0.9998 | 0.9826 | 0.9795 | 0.9689 | 0.9305 | |

P < 0.05 are highlighted in dark cream, 0.05 < P < 0.1 are highlighted in light cream color. Note that the YNAL sample is too small to give relevant results.

## Supplementary Results 2. Daily distance travelled: comparison between species

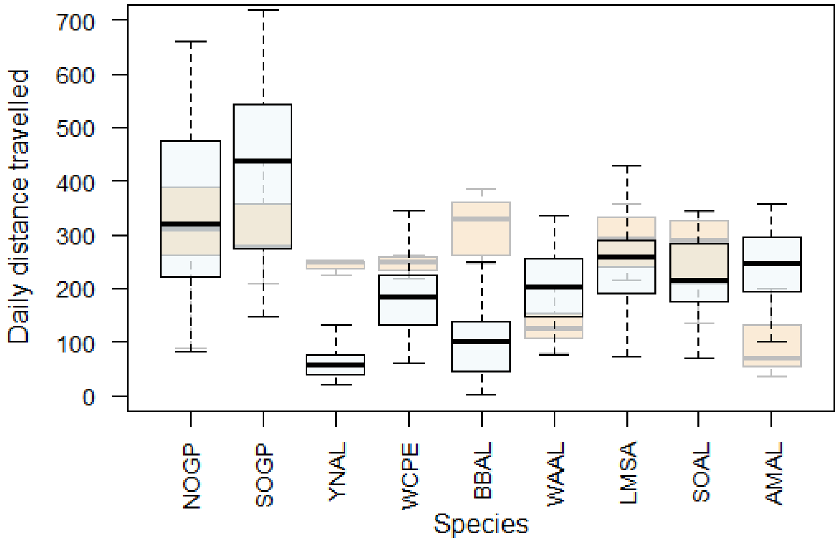


Boxplots of the daily distance travelled by species, during the first 15 days (cream & grey boxes) and averaged over the rest of the trajectories (15 days to 3[rd] month, blue & black boxes). Horizontal bars are the median, box limits are upper and lower quartiles, whiskers represent dispersion.

**Linear mixed model results with the first species as intercept:**

Model: DDT ~ species + random = ~1 | individual

| SPECIES | Value | Std.Error | DF | t-value | p-value |
|---|---|---|---|---|---|
| **(Intercept: NOGP)** | 338.27 | 15.49 | 287 | 21.84 | 0 |
| **SOGP** | 62.39 | 28.08 | 73 | 2.22 | 0.0294 |
| **YNAL** | -231.00 | 34.64 | 73 | -6.67 | 0 |
| **WCPE** | -139.36 | 23.86 | 73 | -5.84 | 0 |
| **BBAL** | -186.16 | 21.48 | 73 | -8.67 | 0 |
| **WAAL** | -153.23 | 19.60 | 73 | -7.82 | 0 |
| **LMSA** | -84.31 | 27.63 | 73 | -3.05 | 0.0032 |
| **SOAL** | -99.74 | 21.58 | 73 | -4.62 | 0 |
| **AMAL** | -121.94 | 21.91 | 73 | -5.57 | 0 |

## Post-hoc Tukey test p-values:

| SPECIES | NOGP | SOGP | YNAL | WCPE | BBAL | WAAL | LMSA | SOAL | AMAL |
|---|---|---|---|---|---|---|---|---|---|
| **NOGP** | | | | | | | | | |
| **SOGP** | 0.3756 | | | | | | | | |
| **YNAL** | <0.01 | <0.01 | | | | | | | |
| **WCPE** | <0.01 | <0.01 | 0.1965 | | | | | | |
| **BBAL** | <0.01 | <0.01 | 0.9254 | 0.5311 | | | | | |
| **WAAL** | <0.01 | <0.01 | 0.3037 | 0.9993 | 0.7197 | | | | |
| **LMSA** | 0.0538 | <0.01 | <0.01 | 0.6084 | <0.01 | 0.1498 | | | |
| **SOAL** | <0.01 | <0.01 | <0.01 | 0.7451 | <0.01 | 0.1133 | 0.9997 | | |
| **AMAL** | <0.01 | <0.01 | 0.04 | 0.9982 | 0.0643 | 0.796 | 0.906 | 0.9817 | |

P < 0.05 are highlighted in dark cream, 0.05 < P < 0.1 are highlighted in light cream colour to highlight the tendency. Note that the YNAL sample is too small to give relevant results.

Sophie de Grissac* [a,b], Luca Börger [c], Audrey Guitteaud [a] and Henri Weimerskirch [a,b]

## Supplementary Table 2. Tukey tests outputs for temporal variation within species in sinuosity and daily distance travelled.

P-value tables of pairwise comparisons between time-periods of 15 days resulting from linear mixed models and post-hoc Tukey test. For each species, the upper part of the table compares sinuosity between time-periods and the lower part of the table compares daily distance travelled (DDT) between time-periods.  $P < 0.05$ (significant effect) are highlighted in dark cream colour. N is the number of individuals for each period. Red text means that the p-value is not relevant and/or prior modelling assumptions were not met due to sample size.

| NOGP | | | SINUOSITY | | | | | | |
|---|---|---|---|---|---|---|---|---|---|
| | month | Days | 1-15 | 16-30 | 31-45 | 46-60 | 61-75 | 76-91 | N |
| DDT | 1 | 1-15 | | 0.999 | 0.727 | 0.000 | 0.020 | 0.197 | 9 |
| | | 16-30 | 0.000 | | 0.917 | 0.002 | 0.061 | 0.377 | 9 |
| | 2 | 31-45 | 0.930 | 0.000 | | 0.048 | 0.437 | 0.902 | 9 |
| | | 46-60 | 0.838 | 0.000 | 1.000 | | 0.948 | 0.630 | 8 |
| | 3 | 61-75 | 0.963 | 0.020 | 0.516 | 0.384 | | 0.984 | 7 |
| | | 76-91 | 1.000 | 0.003 | 0.933 | 0.852 | 0.986 | | 6 |

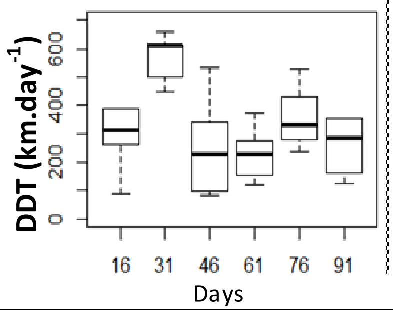


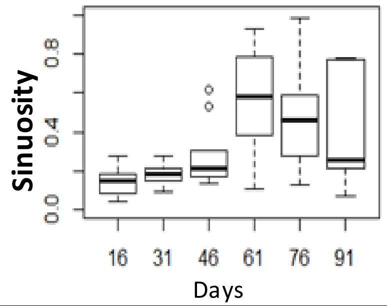


| SOGP | | | SINUOSITY | | | | | | |
|---|---|---|---|---|---|---|---|---|---|
| | month | Days | 1-15 | 16-30 | 31-45 | 46-60 | 61-75 | 76-91 | N |
| DDT | 1 | 1-15 | | 0.142 | 0.246 | 0.965 | 0.619 | 0.008 | 5 |
| | | 16-30 | 0.998 | | 1.000 | 0.935 | 1.000 | 0.538 | 5 |
| | 2 | 31-45 | 0.841 | 0.973 | | 0.975 | 1.000 | 0.416 | 5 |
| | | 46-60 | 0.103 | 0.211 | 0.543 | | 0.986 | 0.187 | 2 |
| | 3 | 61-75 | 0.255 | 0.437 | 0.803 | 0.999 | | 0.563 | 2 |
| | | 76-91 | 0.998 | 1.000 | 0.997 | 0.456 | 0.686 | | 2 |

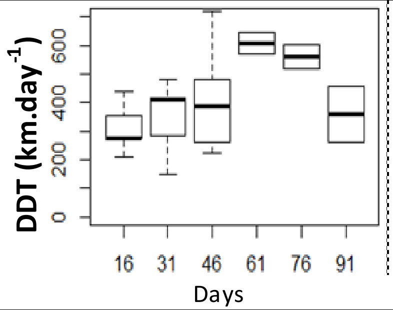


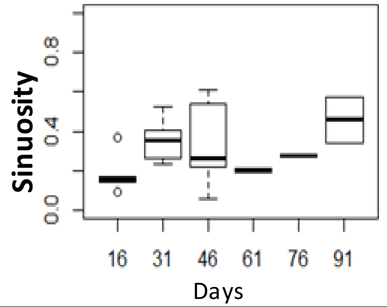


| YNAL | | | SINUOSITY | | | | | | |
|---|---|---|---|---|---|---|---|---|---|
| | month | Days | 1-15 | 16-30 | 31-45 | 46-60 | 61-75 | 76-91 | N |
| DDT | 1 | 1-15 | | 0.227 | 0.142 | 0.611 | 0.138 | 0.904 | 3 |
| | | 16-30 | 0.000 | | 0.998 | 0.999 | 0.950 | 0.995 | 3 |
| | 2 | 31-45 | 0.000 | 0.715 | | 0.969 | 0.996 | 0.960 | 2 |
| | | 46-60 | 0.000 | 0.593 | 1.000 | | 0.864 | 1.000 | 2 |
| | 3 | 61-75 | 0.000 | 0.611 | 0.997 | 1.000 | | 0.857 | 1 |
| | | 76-91 | 0.000 | 0.998 | 0.681 | 0.588 | 0.502 | | 1 |

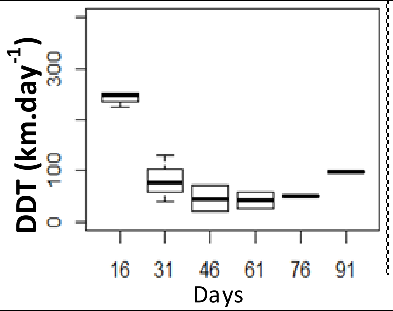


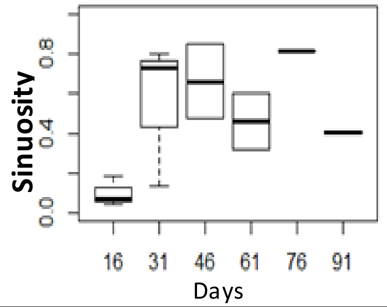

| BBAL | | | SINUOSITY | | | | | | |
|---|---|---|---|---|---|---|---|---|---|
| | month | Days | 1-15 | 16-30 | 31-45 | 46-60 | 61-75 | 76-91 | N |
| DDT | 1 | 1-15 | | 0.019 | 0.000 | 0.000 | 0.000 | 0.000 | 10 |
| | | 16-30 | 0.001 | | 0.487 | 0.222 | 0.380 | 0.008 | 9 |
| | 2 | 31-45 | 0.000 | 0.144 | | 0.997 | 1.000 | 0.476 | 9 |
| | | 46-60 | 0.000 | 0.166 | 1.000 | | 1.000 | 0.757 | 9 |
| | 3 | 61-75 | 0.000 | 0.009 | 0.905 | 0.882 | | 0.645 | 8 |
| | | 76-91 | 0.000 | 0.033 | 0.979 | 0.971 | 1.000 | | 7 |

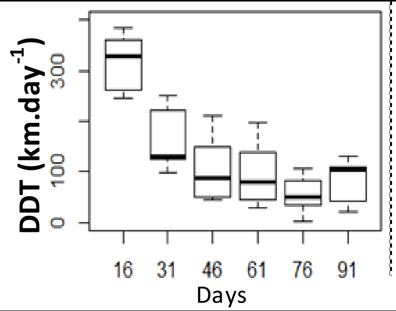


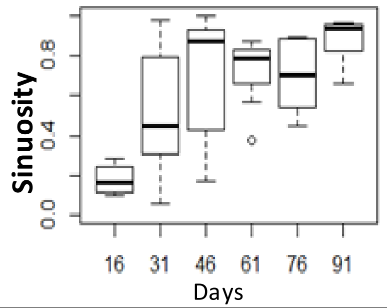


| WCPE | | | SINUOSITY | | | | | | |
|---|---|---|---|---|---|---|---|---|---|
| | month | Days | 1-15 | 16-30 | 31-45 | 46-60 | 61-75 | 76-91 | N |
| DDT | 1 | 1-15 | | 0.185 | 0.425 | 0.004 | 0.000 | 0.001 | 7 |
| | | 16-30 | 0.629 | | 0.997 | 0.676 | 0.210 | 0.322 | 7 |
| | 2 | 31-45 | 1.000 | 0.592 | | 0.401 | 0.079 | 0.147 | 7 |
| | | 46-60 | 0.390 | 0.996 | 0.360 | | 0.979 | 0.990 | 5 |
| | 3 | 61-75 | 0.007 | 0.308 | 0.006 | 0.699 | | 1.000 | 5 |
| | | 76-91 | 0.045 | 0.608 | 0.039 | 0.903 | 1.000 | | 4 |

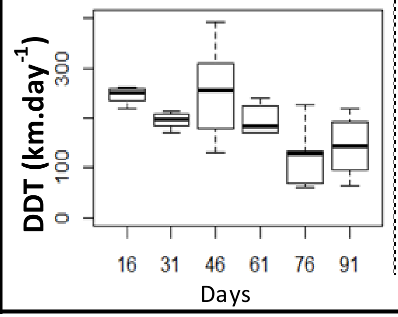


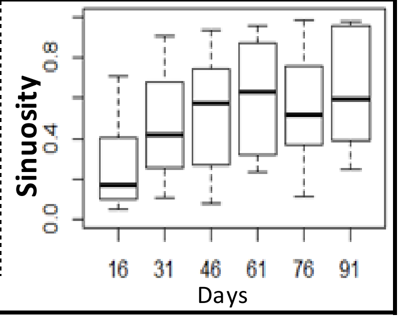


| WAAL | | | SINUOSITY | | | | | | |
|---|---|---|---|---|---|---|---|---|---|
| | month | Days | 1-15 | 16-30 | 31-45 | 46-60 | 61-75 | 76-91 | N |
| DDT | 1 | 1-15 | | 0.359 | 0.016 | 0.001 | 5.65E-02 | 0.001 | 20 |
| | | 16-30 | 0.022 | | 0.920 | 0.421 | 0.9498 | 0.406 | 12 |
| | 2 | 31-45 | 0.006 | 1.000 | | 0.931 | 1 | 0.896 | 15 |
| | | 46-60 | 0.497 | 0.780 | 0.641 | | 0.9554 | 1.000 | 13 |
| | 3 | 61-75 | 0.001 | 0.893 | 0.923 | 0.182 | | 0.923 | 10 |
| | | 76-91 | 0.202 | 0.993 | 0.978 | 0.987 | 0.6162 | | 10 |

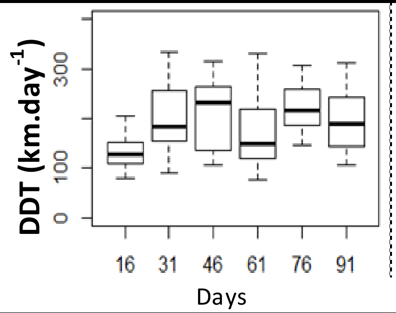


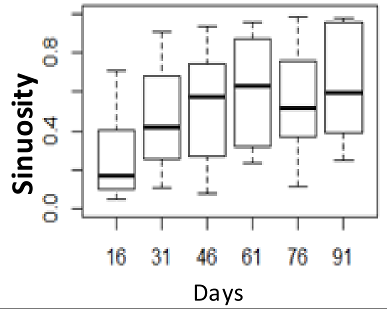

| LMSA | | | SINUOSITY | | | | | | |
|---|---|---|---|---|---|---|---|---|---|
| | **month** | **Days** | **1-15** | **16-30** | **31-45** | **46-60** | **61-75** | **76-91** | **N** |
| **DDT** | **1** | **1-15** | | 0.446 | 0.669 | 0.998 | 0.995 | 0.646 | 5 |
| | | **16-30** | 0.999 | | 1.000 | 0.819 | 0.971 | 0.993 | 6 |
| | **2** | **31-45** | 1.000 | 1.000 | | 0.914 | 0.987 | 0.990 | 4 |
| | | **46-60** | 0.137 | 0.038 | 0.106 | | 1.000 | 0.812 | 4 |
| | **3** | **61-75** | 0.012 | 0.004 | 0.009 | 0.785 | | 0.921 | 2 |
| | | **76-91** | 0.011 | 0.004 | 0.011 | 0.426 | 0.974 | | 1 |

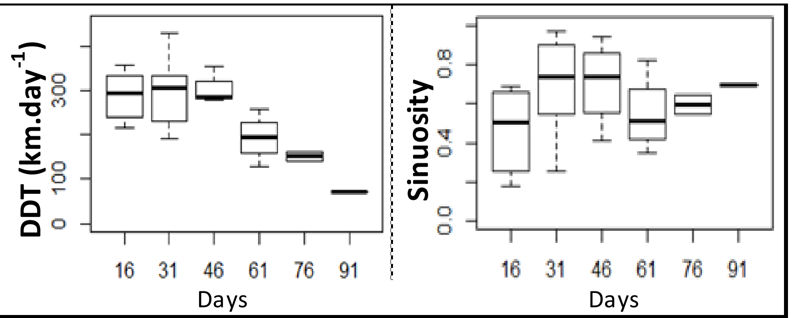


| SOAL | | | SINUOSITY | | | | | | |
|---|---|---|---|---|---|---|---|---|---|
| | **month** | **Days** | **1-15** | **16-30** | **31-45** | **46-60** | **61-75** | **76-91** | **N** |
| **DDT** | **1** | **1-15** | | 0.981 | 1.000 | 1.000 | 1.000 | 0.981 | 10 |
| | | **16-30** | 0.580 | | 0.981 | 0.976 | 0.944 | 0.755 | 10 |
| | **2** | **31-45** | 0.997 | 0.282 | | 1.000 | 1.000 | 0.980 | 10 |
| | | **46-60** | 0.790 | 1.000 | 0.516 | | 1.000 | 0.994 | 7 |
| | **3** | **61-75** | 0.596 | 1.000 | 0.322 | 1.000 | | 0.999 | 7 |
| | | **76-91** | 0.403 | 0.998 | 0.182 | 0.994 | 1.000 | | 7 |

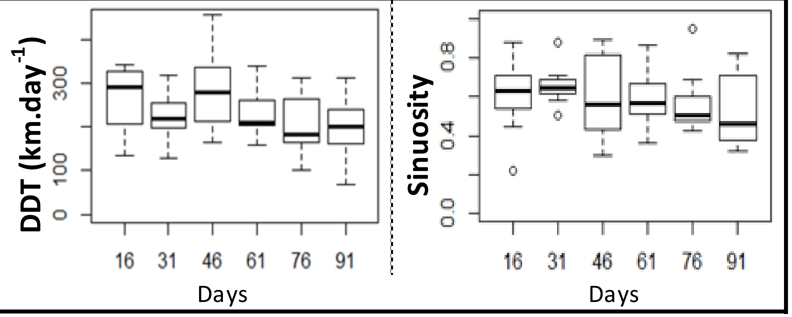


| AMAL | | | SINUOSITY | | | | | | |
|---|---|---|---|---|---|---|---|---|---|
| | **month** | **Days** | **1-15** | **16-30** | **31-45** | **46-60** | **61-75** | **76-91** | **N** |
| **DDT** | **1** | **1-15** | | 0.927 | 1.000 | 0.978 | 0.200 | 0.881 | 9 |
| | | **16-30** | 0.000 | | 0.961 | 1.000 | 0.742 | 1.000 | 9 |
| | **2** | **31-45** | 0.000 | 0.208 | | 0.991 | 0.276 | 0.927 | 8 |
| | | **46-60** | 0.000 | 0.159 | 1.000 | | 0.640 | 0.999 | 8 |
| | **3** | **61-75** | 0.000 | 0.000 | 0.284 | 0.351 | | 0.875 | 7 |
| | | **76-91** | 0.000 | 0.066 | 0.993 | 0.998 | 0.666 | | 7 |

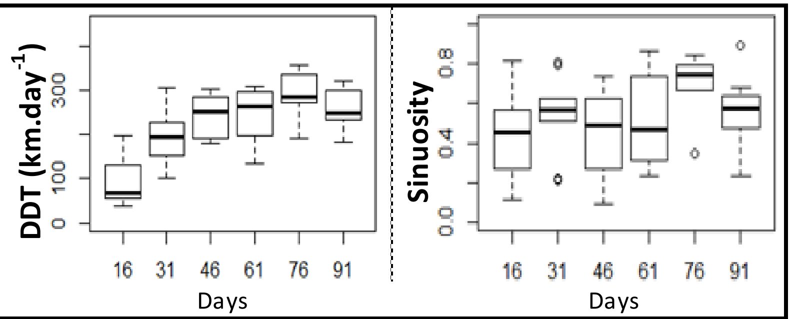

# Supplementary Table.3. Distance to colony: comparison between species

## Linear mixed model results with northern giant petrel (NOGP) as intercept:

Model: log($Range_{1month}$) ~ species + random = ~1 | individual

| SPECIES | Value | Std.Error | DF | t-value | p-value |
|---|---|---|---|---|---|
| **(Intercept: NOGP)** | 9.33 | 0.136 | 61 | 68.60 | 0 |
| **SOGP** | -0.40 | 0.228 | 61 | -1.76 | 0.0835 |
| **YNAL** | -1.02 | 0.272 | 61 | -3.76 | 0.0004 |
| **WCPE** | -1.38 | 0.206 | 61 | -6.70 | 0 |
| **BBAL** | -0.76 | 0.192 | 61 | -3.95 | 0.0002 |
| **WAAL** | -1.71 | 0.180 | 61 | -9.51 | 0 |
| **LMSA** | -1.59 | 0.215 | 61 | -7.41 | 0 |
| **SOAL** | -1.71 | 0.187 | 61 | -9.10 | 0 |
| **AMAL** | -2.04 | 0.192 | 61 | -10.60 | 0 |

The response variable, i.e. the maximum distance to colony reach during the first month, was log-transformed to meet the model assumptions.

## Supplementary Fig. 2. Juveniles’ habitat characterisation.

Boxplot of bathymetry (a), SST (a) and Chlorophyll *a* (c) along the tracks of juveniles of the nine species with median (bold line), interquartile range (boxes), dispersion (vertical lines) and outliers (points). Red lines and stars indicate Tukey test pairwise comparisons P < 0.01 (***) or P < 0.05 (**) between species.

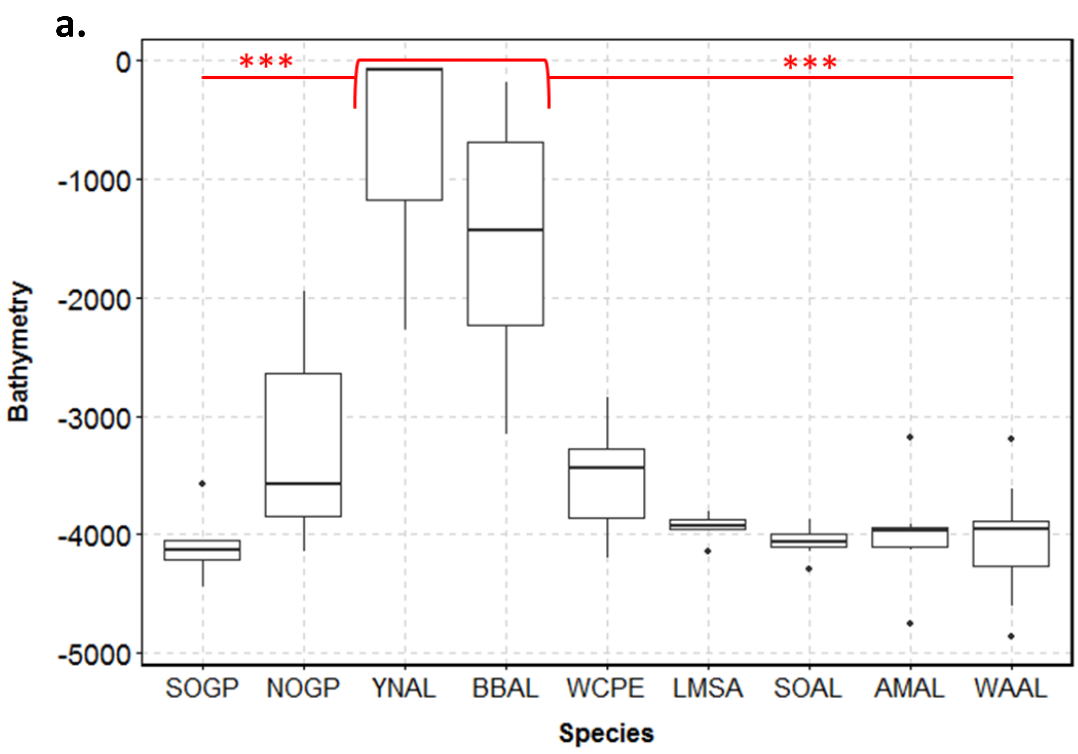

b.

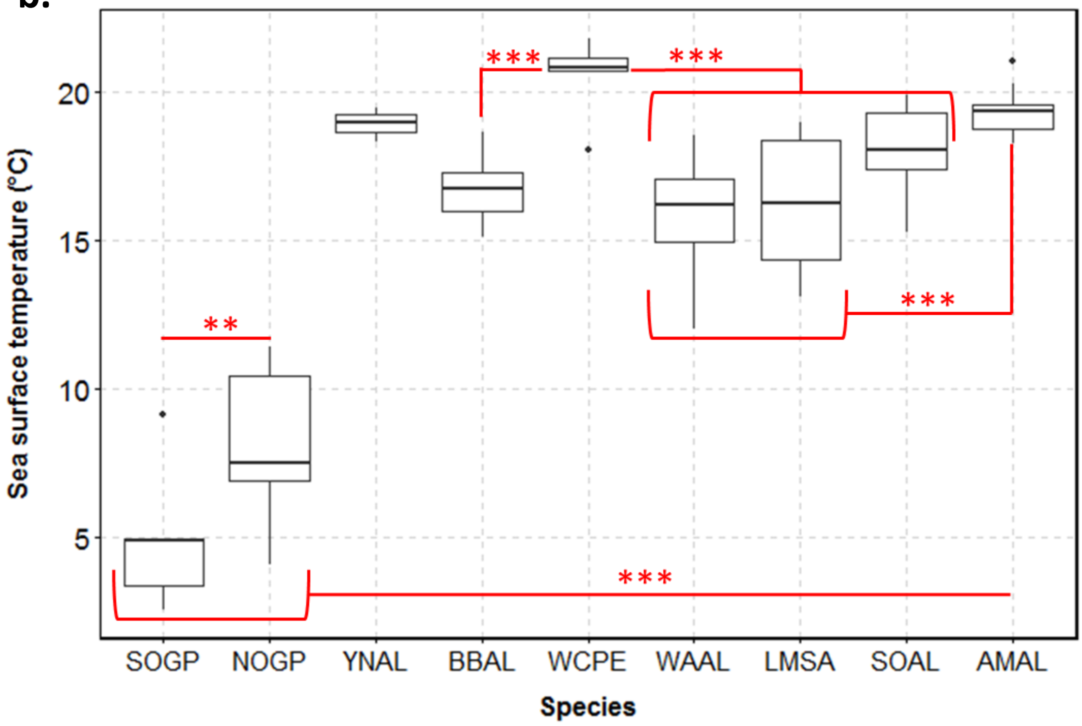
Sea surface temperature (°C)
20
15
10
5
***
***
**
***
***
SOGP
NOGP
YNAL
BBAL
WCPE
WAAL
LMSA
SOAL
AMAL
Species

c.

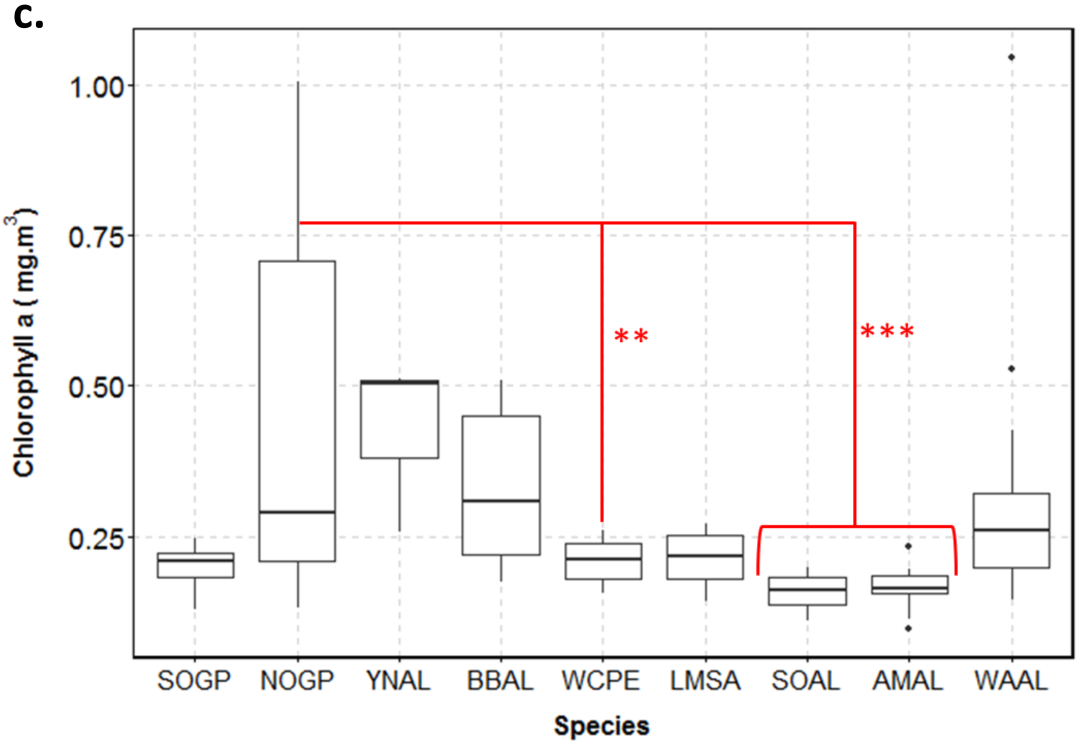
Chlorophyll a ( mg.m$^{3}$)
1.00
0.75
0.50
0.25
**
***
SOGP
NOGP
YNAL
BBAL
WCPE
LMSA
SOAL
AMAL
WAAL
Species
